# A Viral microRNA Cluster Regulates the Expression of PTEN, p27 and of a bcl-2 Homolog

**DOI:** 10.1371/journal.ppat.1005405

**Published:** 2016-01-22

**Authors:** Katharina Bernhardt, Janina Haar, Ming-Han Tsai, Remy Poirey, Regina Feederle, Henri-Jacques Delecluse

**Affiliations:** 1 Pathogenesis of Virus Associated Tumors, German Cancer Research Center, Heidelberg, Germany; 2 Inserm unit U1074, Heidelberg, Germany; Tulane Health Sciences Center, UNITED STATES

## Abstract

The Epstein-Barr virus (EBV) infects and transforms B-lymphocytes with high efficiency. This process requires expression of the viral latent proteins and of the 3 miR-BHRF1 microRNAs. Here we show that B-cells infected by a virus that lacks these non-coding RNAs (Δ123) grew more slowly between day 5 and day 20, relative to wild type controls. This effect could be ascribed to a reduced S phase entry combined with a moderately increased apoptosis rate. Whilst the first phenotypic trait was consistent with an enhanced PTEN expression in B-cells infected with Δ123, the second could be explained by very low BHRF1 protein and RNA levels in the same cells. Indeed, B-cells infected either by a recombinant virus that lacks the BHRF1 protein, a viral bcl-2 homolog, or by Δ123 underwent a similar degree of apoptosis, whereas knockouts of both BHRF1 microRNAs and protein proved transformation-incompetent. We find that that the miR-BHRF1-3 seed regions, and to a lesser extent those of miR-BHRF1-2 mediate these stimulatory effects. After this critical period, B-cells infected with the Δ123 mutant recovered a normal growth rate and became more resistant to provoked apoptosis. This resulted from an enhanced BHRF1 protein expression relative to cells infected with wild type viruses and correlated with decreased p27 expression, two pro-oncogenic events. The upregulation of BHRF1 can be explained by the observation that large BHRF1 mRNAs are the source of BHRF1 protein but are destroyed following BHRF1 microRNA processing, in particular of miR-BHRF1-2. The BHRF1 microRNAs are unlikely to directly target p27 but their absence may facilitate the selection of B-cells that express low levels of this protein. Thus, the BHRF1 microRNAs allowed a time-restricted expression of the BHRF1 protein to innocuously expand the virus B-cell reservoir during the first weeks post-infection without increasing long-term immune pressure.

## Author Summary

This paper explains some of the molecular mechanisms used by the Epstein-Barr virus (EBV) BHRF1 microRNA cluster to enhance transformation of B-cells after infection. We find that B-cells exposed to a virus that lacks the BHRF1 microRNAs (Δ123) undergo more apoptosis and grow more slowly between the second and the fourth weeks after infection than cells infected by an intact virus. These effects are partly mediated by the viral protein BHRF1, a homolog of the anti-apoptotic bcl-2 protein. The viral microRNAs allow abundant expression of BHRF1 early after infection and its down-regulation when transformation has been established. The first effect is mediated by the seed regions of miR-BHRF1-2 and -3, whereas the second is dependent on RNA cleavage mediated by processing of miR-BHRF1-2. Furthermore, we found that the ability of the BHRF1 microRNAs to increase cell cycle entry is related to their ability to downregulate PTEN, a crucial negative regulator of the cell cycle. We also study the consequences of the absence of the microRNAs for the infected cells. B-cells infected with Δ123 become more resistant to apoptosis and express lower levels of p27, two events that facilitate the development of genome instability. Thus, the viral microRNAs allow rapid and innocuous expansion of infected B-cells, their long-term reservoir, thereby facilitating the life-long coexistence between the virus and its host.

## Introduction

The Epstein-Barr virus (EBV) is the first discovered tumor human virus and is etiologically associated with approximately 2% of all tumors worldwide [1, 2]. These tumors are largely diverse in terms of lineage and include multiple types of lymphomas and carcinomas [3]. Immune deficiency, e.g. caused by immunosuppressive regimen is a strong risk factor for the development of EBV-associated lymphomas [2]. These tumors are thought, at least to some extent, to reflect EBV’s ability to transform primary B-cells [2]. This process can be easily observed *in vitro* as it leads to the establishment of lymphoblastoid cell lines (LCLs) and requires the simultaneous expression of some members of the viral latent gene family [2]. In recent years, it has become clear that the BHRF1 microRNAs (miRNAs) encoded by the virus markedly potentiate this process. Recombinant viruses that lack one or several of these three miR-BHRF1s are less transforming than their wild type counterparts and the effect is cumulative [4–6]. One study has ascribed this property to the ability of the BHRF1 miRNAs to prevent massive apoptosis in the first days of infection [4]. Furthermore, viruses that lack the three BHRF1 miRNAs (Δ123) grow more slowly and display abnormalities of the cell cycle [4, 5]. Humanized NSG mice infected by Δ123 eventually develop B-cell proliferations that are indistinguishable from those caused by wild type infection, but cell growth induced by the mutant is delayed by several weeks, confirming that the BHRF1 miRNAs are particularly required in the early phases of infection [7]. The BHRF1 protein, around which the BHRF1 microRNAs are located, has also been implicated in EBV-mediated B-cell transformation, although its role appears to be more difficult to define. BHRF1 is a bcl-2 homolog that shares its anti-apoptotic properties [8, 9]. Although its expression level is hardly detectable in LCLs, it is strongly expressed in the Wp-restricted Burkitt’s lymphoma (BL) cells, a subset of Burkitt’s lymphomas that are infected by EBVs that carry a deletion of the EBNA2 gene and whose latent genes are driven by the Wp promoter [10, 11]. A recombinant virus that lacks the BHRF1 protein retains full transformation abilities, suggesting that this protein is dispensable for transformation [12]. However, its enhanced expression in Wp-restricted BLs leads to a markedly enhanced resistance to apoptosis induced by ionomycin [11, 13]. Thus, both the BHRF1 protein and the BHRF1 miRNAs have been implicated in the regulation of apoptosis.

The prominent role played by some of the EBV latent genes in B-cell transformation raises the question of a possible interaction of the BHRF1 miRNAs with the latent genes. Although these have not been directly identified in a search for the miR-BHRF1 targets, we have clearly identified the EBNA-LP latent gene as a, probably indirect, target of the BHRF1 miRNAs [5]. The expression of this protein is usually downregulated in LCLs after several weeks of growth in culture but this process is largely delayed after infection with Δ123 [5]. The other latent genes were also upregulated in LCLs infected by Δ123 relative to wild type counterparts but the effect was much weaker and inconstant. This raised the question whether the BHRF1 open reading frame is also a target of the BHRF1 miRNAs but its transcription level was not affected in cells infected by the mutant [4, 5].

In this paper we examine the role played by the complete BHRF1 locus during EBV infection. We found that the BHRF1 miRNA cluster controls the temporal expression of the BHRF1 protein but also downregulates PTEN. The deletion of this cluster also led to the frequent emergence of transformed B-cells with a downregulation of p27.

## Results

### Phenotypic traits of B-cells infected with Δ123

**Fig 1:**
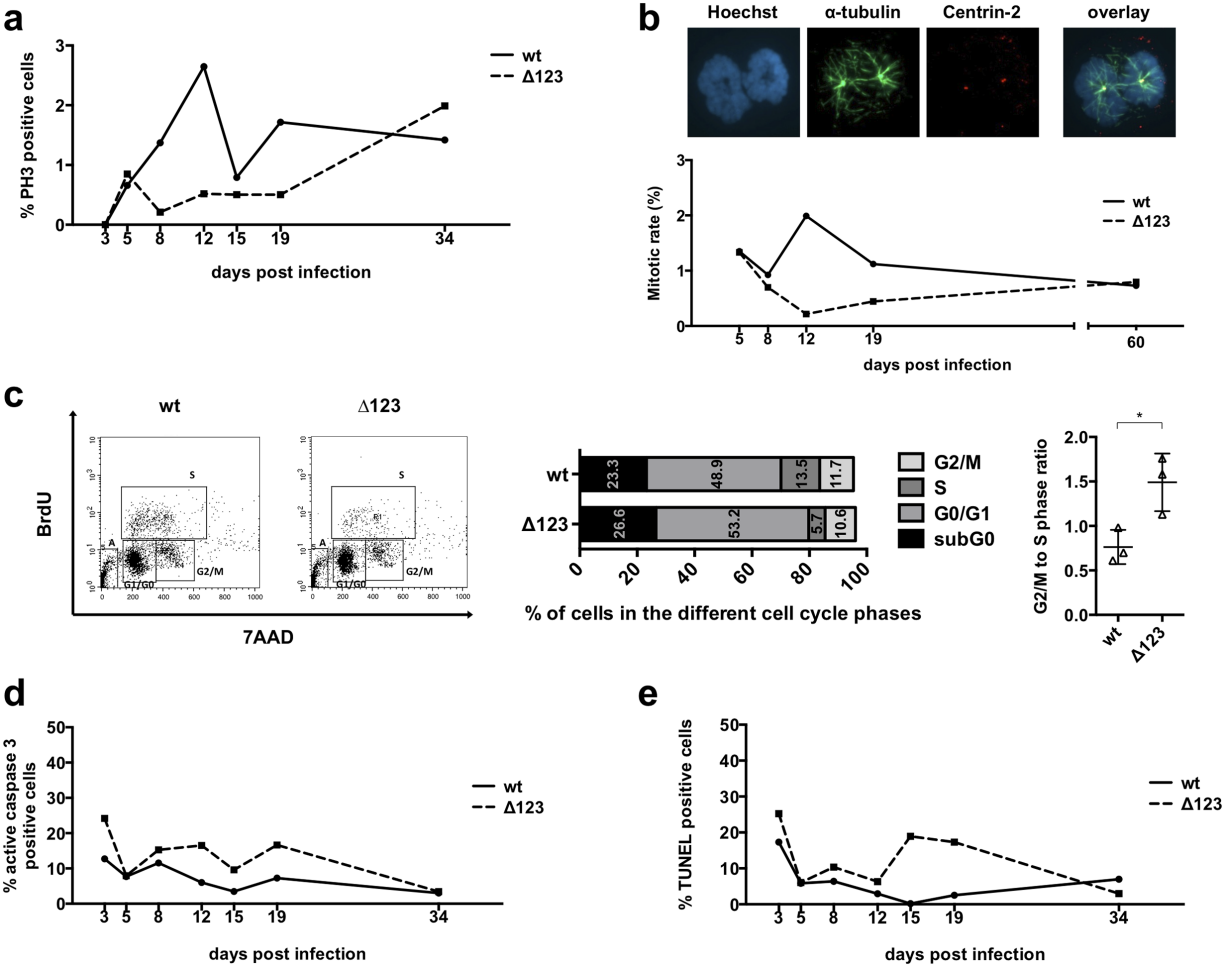
Phenotypic traits of B-cells infected with Δ123. Primary B-cells were infected with B95-8 wild type (wt) or a recombinant virus lacking the three BHRF1 miRNAs (Δ123) and monitored between day 3 and 34 after infection. The mitotic rate was analyzed by immunofluorescence staining of phospho-histone H3 (PH3) (a), or Hoechst 33258 staining of DNA combined with a Centrin-2 (Cy3) and α-tubulin (Alexa488) double staining (b). BrdU incorporation assays were performed at day 13 to compare the cell cycle distribution of B-cells transformed with wt virus or the Δ123 mutant (c). The adjacent 2 graphs give the percentage of cells present in the different phases of the cell cycle as well as the ratio of cells in the G2/M and in the S phase, based on the results of the BrdU assay performed on three blood samples. We determined the degree of apoptosis in infected B-lymphocytes by immunofluorescence staining of active caspase 3 (d) or using a TUNEL assay at the indicated time points after infection (e). All experiments were repeated with cells from three different B-cell donors.

EBV infection of B-cells induces permanent cell division that gives rise to the establishment of lymphoblastoid cell lines (LCLs). Therefore, we began our investigations by monitoring cell growth and cell vitality over the first four weeks after infection with the Δ123 mutant or with wild type EBV controls. This was achieved by directly counting mitoses in the samples or staining cells with phospho-histone H3, a marker of cells undergoing mitosis. Both methods showed no evidence of cell division before day 3, as expected [14]. Cells infected with the wild type control then began dividing, reaching a peak at around day 10 and then maintained a constant mitotic rate between 1 and 2% (Fig 1a and 1b). The same B-cells infected in parallel with Δ123 differed from wild type controls in that their mitotic rate was 2–3 fold lower. However, after 25 days, both mutants and controls hardly showed any difference. These data suggested transient differences in cell cycle regulation between both groups of cells. Therefore, we performed a BrdU incorporation assay at day 13 post-infection (p.i.). This experiment showed a decrease in the fraction of cells that entered the S phase, as well as a relative increase in the number of cells present in G2/M in Δ123-infected cells (Fig 1c). This resulted in a statistically significant difference in the G2/M to S ratio between B-cells infected with Δ123 or with wild type virus. We then stained the same infected cells with an antibody specific to cleaved pro-caspase 3 that detects the form of the protein activated during apoptosis, coupled to a TUNEL assay that detects double strand DNA breaks. These assays are well suited for the detection of apoptosis at the single cell level and the results are summarized in Fig 1d and 1e. They showed that between day 1 and day 5, B-cells infected with wild type viruses or with Δ123 behaved identically. However, from day 8 on, the apoptosis rate grew larger in the cells infected with the miRNA triple mutant and became twice as high at day 15. The apoptotic rate decreased in these cells after day 18 to reach those evinced by wild type LCLs at day 34. Altogether, we conclude that cells infected by the Δ123 mutant do not enter the cell cycle as efficiently and undergo more apoptosis than the controls after initiation of cell division between day 8 and 20 after infection. As described in the sequel, transformation of additional B-cell samples revealed that the amplitude of the difference between B-cells infected by wild type or mutant viruses in terms of apoptotic rate and mitotic growth can vary. However, the general picture remained similar.

### B-cells freshly infected with EBV strongly express BHRF1

**Fig 2:**
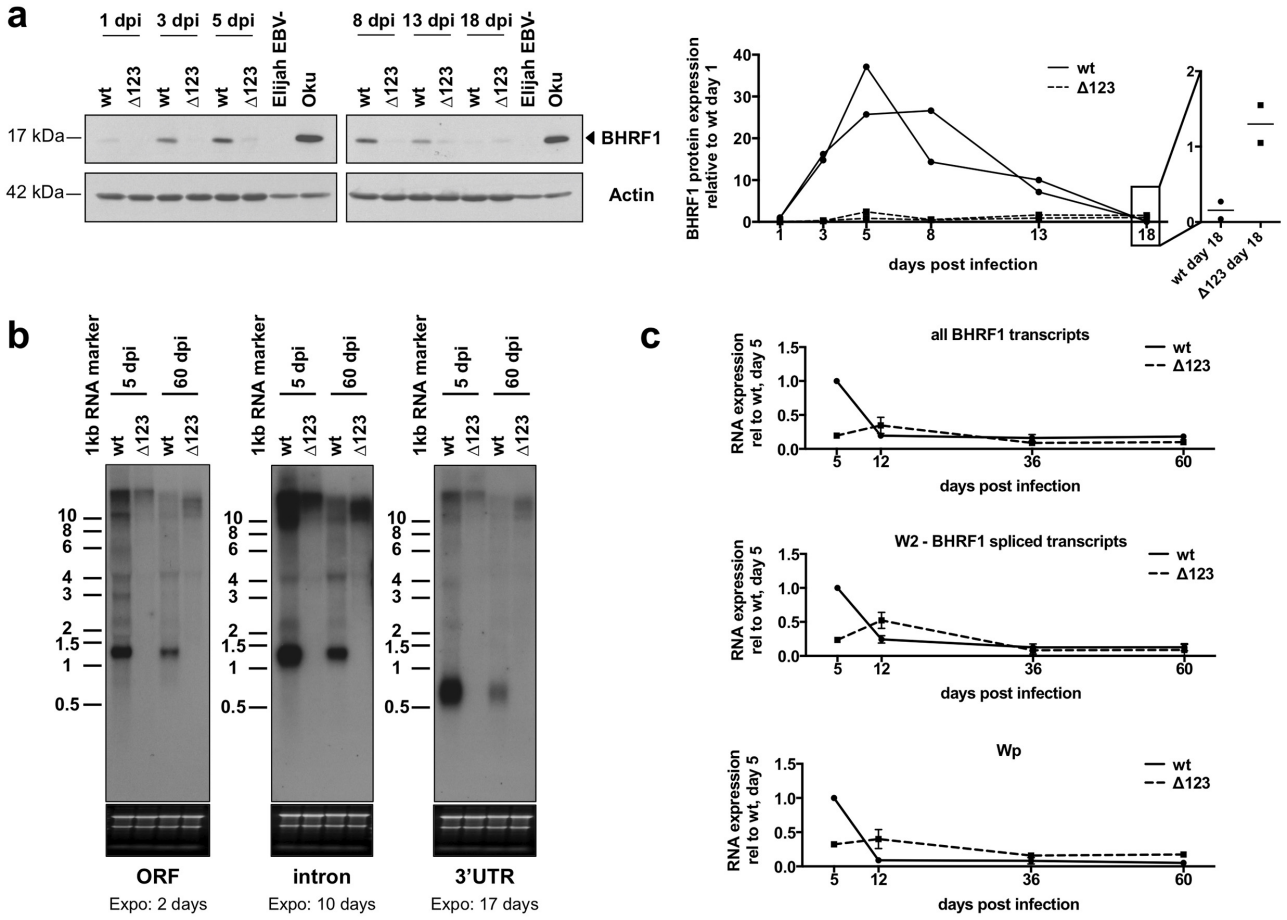
B-cells freshly infected with EBV strongly express BHRF1. (a) B-cells were infected with EBV wild type or the miR-BHRF1-negative virus (Δ123) and harvested at the indicated time points post-infection for immunoblotting with a BHRF1-specific antibody. Actin served as a loading control. The EBV negative Elijah BL cell line and the Wp-restricted Oku-BL served as negative and positive controls for BHRF1 expression, respectively. Actin levels in the two BL lines were repeatedly found to be lower than in LCLs, although the same amount of total protein was loaded for each sample. The right panel shows an ImageJ-based quantification of BHRF1 protein expression normalized for actin with a close-up view on day 18 p.i. Protein expression is shown relative to BHRF1 levels in the LCL transformed by wt EBV at day 1 post-infection. Results of two independent infections with unrelated B-cell samples are shown. (b) The picture shows a northern blot analysis of LCLs generated with wild type virus or Δ123 at day 5 or 60 post-infection (dpi). The blots were hybridized with three probes that span the open reading frame (ORF) BHRF1 gene, its 3’UTR and its intron. The exposure times are indicated below the blots (c) Three B-cell samples from independent donors infected with wild type EBV or Δ123 were collected at day 5, 12, 36 and 60 post-infection. The graph shows the evolution of the BHRF1 RNA expression levels in these cells over time.

The BHRF1 miRNA cluster is located around the BHRF1 gene, whose protein product is endowed with anti-apoptotic properties [9, 11]. Therefore, we monitored BHRF1 protein expression by western blot around the critical period, between 1 and 20 days p.i. We used an EBV-negative clone of the Burkitt’s lymphoma cell line Elijah and the Wp-restricted cell line Oku as a negative and a positive control, respectively. This assay, shown in Fig 2a, revealed that the BHRF1 protein is transiently produced in cells infected by the wild type controls at levels in the range of those observed in Oku. Oku is known to express BHRF1 at much higher levels than established LCLs [11]. Expression began at day 1, became fully visible at day 3 and reached a peak at day 5, after which it decreased again to nearly disappear at day 18. In stark contrast, B-cells infected with Δ123 produced hardly detectable levels of the protein. We then performed northern blots at the peak of BHRF1 protein expression at day 5 in B-cells infected with EBV wild type or Δ123 (Fig 2b). This assay revealed that B-cells infected with wild type viruses produce multiple and abundant transcripts ranging from 1.3 to larger than 10 kb. In comparison, cells infected with Δ123 showed only large transcripts that were altogether much less abundant than in the wild type-infected LCLs. Thus, the reduced BHRF1 protein expression correlates with reduced BHRF1-specific transcription. We left the infected cells grow for another 55 days and repeated the experiment. The blots revealed that by that time the 1.3 kb band had become prominent in the LCL infected by wild type viruses with larger bands becoming much fainter. Interestingly, the LCL infected with Δ123 retained large BHRF1 transcripts, albeit slightly smaller than at day 5. Consequently, the large BHRF1 transcripts were more abundant in LCLs infected with Δ123 than in those generated with wild type controls. These results indicate that the wild type BHRF1 locus is transcribed at a different rate at an early and late stage of infection. However, whilst transcription markedly diminished with time in the wild type LCL, it remained nearly constant in cells infected with the mutant. We attempted to confirm these investigations by qPCR. We interrogated several BHRF1 transcripts, as indicated in Fig 2c, as well as those driven by the Wp promoter that dominates transcription at day 5. We found that LCLs infected with the wild type virus express much higher levels of BHRF1 transcripts at day 5 than at day 12. The same pattern was visible for W2-BHRF1 spliced transcripts and for Wp-driven transcripts. In the LCL infected with the Δ123 mutant, the BHRF1 transcripts at day 5 were expressed at a much lower level (10 to 20% of wild type level). They then increased to become more abundant than in the wild type controls, to fall again at approximately wild type levels after one month of infection. Thus, the usual pattern of Wp transcription, i.e. high levels shortly after infection that rapidly decrease after a few days, is translated to the right and reduced in its initial peak in cells infected with the mutant. We conclude from these findings that the BHRF1 locus undergoes very dynamic changes in terms of transcription over time and that BHRF1 transcription and translation is abnormally low at an early time point in LCLs infected by Δ123.

**Fig 3:**
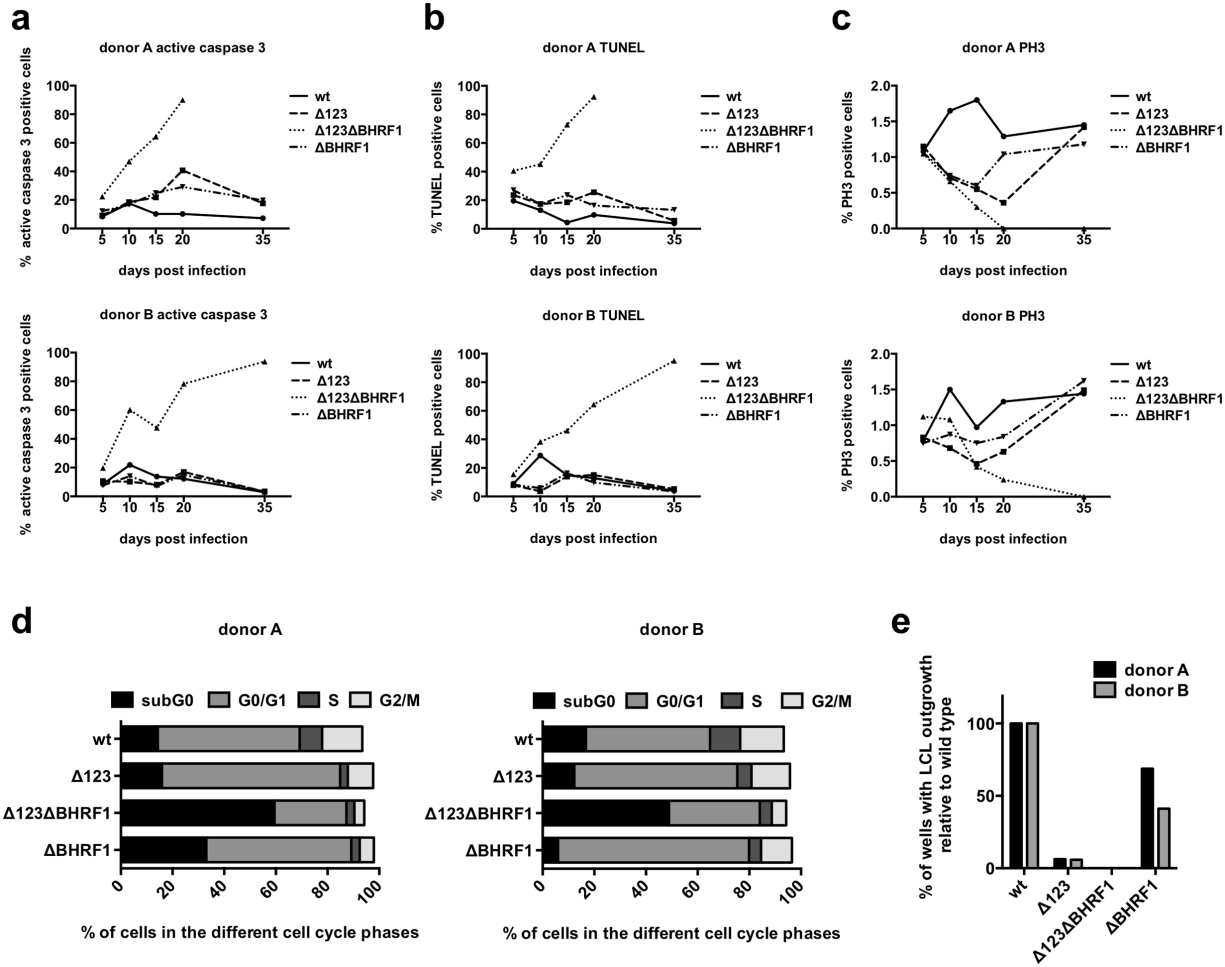
The reduced BHRF1 expression in B-cells infected with Δ123 leads to a moderately increased apoptosis. Primary B-cells were infected with wild type, Δ123, ΔBHRF1 or Δ123ΔBHRF1 and monitored over time by immunofluorescence staining of active caspase 3 (a), by TUNEL assay (b), and by phospho-histone H3 staining (PH3) (c). Two out of five blood samples are shown. BrdU incorporation on day 14 was determined in B-cells transformed with wild type, Δ123, ΔBHRF1 and Δ123ΔBHRF1 (d). The results obtained with two samples are shown. Transformation assays were performed by infecting primary B-cells with 0.01 infectious virus per cell. 500 cells per well were seeded on 96-well cluster plates coated with 50Gy irradiated WI38 feeder cells and the number of wells with LCL outgrowth was determined 30 days after infection (e).

The latter results also suggested that the low abundance of the BHRF1 protein might be responsible for the observed increased apoptosis rate. We addressed this issue by studying the phenotype of a virus that carries an inactivating point mutation of the BHRF1 start codon (ΔBHRF1, see S1 Fig) and of a virus that lacks both the BHRF1 miRNAs and the BHRF1 open reading frame (Δ123ΔBHRF1). B-cells exposed to these viruses or to a wild type control showed little differences in apoptosis and cell growth until day 5 (Fig 3a, 3b and 3c). From day 6 onwards, apoptosis remained low in B-cells infected with wild type EBV but increased steadily in B-cells infected with the Δ123ΔBHRF1 double mutant to reach 100% between day 15 and day 30, depending on the blood samples tested. B-cells transformed by wild type viruses retained a mitotic rate of about 1.5% of the total cells after staining with PH3. In contrast, LCLs generated with the Δ123ΔBHRF1 double mutant exhibited a much lower mitotic rate that never exceeded 0.5%. LCLs transformed with ΔBHRF1 or Δ123 showed an intermediate profile between these 2 extreme phenotypes. In some of the 5 studied cases, the ΔBHRF1 LCLs and the Δ123-infected LCLs did not differ markedly from wild type-infected LCLs, with a good mitotic rate and a low level of apoptosis. In others, their behavior was closer to those of cells infected with the double knockout virus. In all cases, the LCLs generated with the ΔBHRF1 virus displayed higher mitotic rates than their Δ123 counterparts. We addressed this issue in more detail and performed a BrdU incorporation assay early after infection with wild type EBV, Δ123 and ΔBHRF1. The results of this experiment are depicted in Fig 3d and show that the percentage of cells in S phase is lowest in B-cells transformed with Δ123, highest in those transformed with wild type EBV and intermediate in cells transformed with ΔBHRF1. These data suggest that the mild increase in apoptosis and some of the cell cycle abnormalities observed in B-cells infected with Δ123 could be largely explained by the reduction in BHRF1 protein levels and that the low BHRF1 expression level, the only difference between B-cells infected with Δ123 and those infected with Δ123ΔBHRF1, becomes indispensable for survival of cells infected by a Δ123 virus.

We then performed transformation assays at low cell density and low MOI on feeder cells in 96-well cluster plates (Fig 3e). Feeder cells have previously been shown to reduce apoptosis in EBV-infected B-cells [15]. This assay showed that the B-cell transformation rate was highest in cells infected with wild type EBV, whilst B-cells infected with the Δ123ΔBHRF1 double knockout did not show any signs of outgrowth. The transformation assays performed with ΔBHRF1 or Δ123 showed again intermediate results. However, the transformation rate was much higher after infection with ΔBHRF1 than after infection with Δ123. Thus, the absence of BHRF1 but not those of the BHRF1 miRNAs can be largely compensated by feeder cells. Therefore, the Δ123 phenotype is not limited to a reduction in BHRF1 levels. This experiment also shows that ΔBHRF1 viruses are mildly less transforming than wild type viruses, an observation consistent with the moderate reduction in S phase entry observed previously. We tested whether it is possible at all to generate LCLs with the Δ123ΔBHRF1 double mutant. To this end, we infected B-cells from 4 different donors at high cell density (10^4^ cells per well of a 96-well cluster plate) at an MOI of 10 infectious units per cell and kept the cells on a feeder cell layer for 1.5 months. This led to the establishment of LCLs in 2 out of 4 cases.

### miR-BHRF1-3 and miR-BHRF1-2 stimulate BHRF1 transcription and translation early after infection

**Fig 4:**
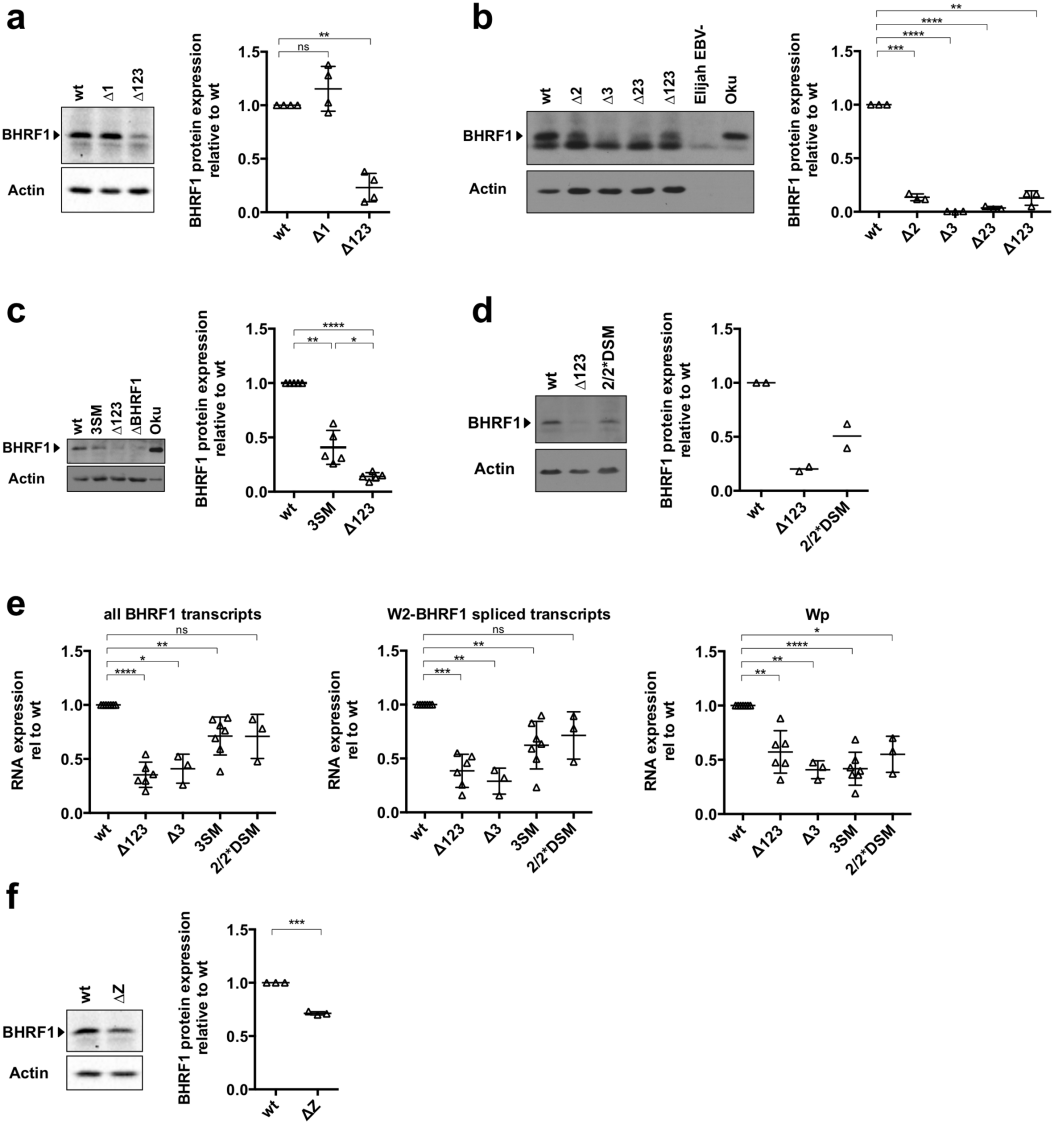
miR-BHRF1-3 and miR-BHRF1-2 stimulate BHRF1 transcription and translation early after infection. BHRF1 protein expression at day 5 after infection of primary B-cells was determined by western blotting for cells infected with wild type, Δ123 and Δ1 (a), Δ2, Δ3, and Δ23 (b), 3SM and ΔBHRF1 (c), and 2/2*DSM (d). The figure shows a representative western blot and the adjacent graphs relative intensities of the obtained signals after quantification with ImageJ. (e) BHRF1 and Wp-driven transcripts were quantified by qPCR 5 days after B-cell infection with wt, Δ123, Δ3, 2/2*DSM and 3SM. The graph gives the results relative to values observed in cells infected with the wild type virus. (f) BHRF1 expression in cells infected with a replication-incompetent ΔZ mutant. One representative blot is shown together with the ImageJ-based quantification of multiple B-cell donors. The results are given as a ratio of signals recorded for LCLs infected with the mutant and the wt virus and are normalized for differences in actin expression.

The results obtained with the Δ123 virus showed that the BHRF1 miRNAs are involved in the control of the BHRF1 protein production. We determined which of these miRNAs is implicated in this process by infecting primary B-cells with viruses that lack one of the BHRF1 miRNAs. Western blot analysis with a BHRF1-specific antibody revealed that infection of primary B-cells with a virus that lacks miR-BHRF1-1 or with the wild type control gave rise to the same level of BHRF1 protein production at day 5 (Fig 4a). In contrast, BHRF1 expression in B-cells infected with single and double miRNA mutants that lack miR-BHRF1-2, miR-BHRF1-3 or both (Δ2, Δ3, Δ23) was markedly reduced relative to B-cells infected with wild type viruses (Fig 4b and S1 Fig). We wished to confirm these findings by infecting B-cells with a virus that carries a seed mutation in miR-BHRF1-3 (3SM) (S1 Fig). This mutant expresses miR-BHRF1-1 and miR-BHRF1-2 at wild type levels (S2 Fig). Primary B-cells were infected in parallel with wild type virus, Δ123 and 3SM and harvested at day 5 post-infection. We performed an immunoblot with an anti-BHRF1 antibody that confirmed a clearly decreased BHRF1 protein expression after infection with 3SM, relative to wild type levels, although the amplitude of the effect was not as pronounced as after infection with the Δ3 virus (Fig 4c). This might point towards a role for miR-BHRF1-3* in this process. However, the low expression of this miRNA in LCLs argues against its role in the regulation of BHRF1 protein expression [16, 17]. We also performed this experiment with a mutant that carries mutations of both seed regions encoded by pre-miR-BHRF1-2 (2/2*DSM) (S1 Fig). Indeed, we previously showed that the Δ2 virus also evinces a reduced miR-BHRF1-3 expression [6]. 2/2*DSM expresses normal miR-BHRF1-3 levels and is therefore suitable to study the contribution of miR-BHRF1-2 to the regulation of BHRF1 protein expression (S2 Fig). B-cells infected with this mutant displayed no altered phenotype and expressed the BHRF1 protein at day 5 post-infection at approximately 60% of the levels observed in cells infected with wild type viruses (Fig 4d). Altogether, this set of experiments identify miR-BHRF1-3, and to a lesser extent miR-BHRF1-2, seed regions as positive modulators of BHRF1 protein expression early after infection. We then gauged the expression of the BHRF1 transcripts at the same early time point using qPCR (Fig 4e). This assay confirmed the reduced Wp-driven transcription in B-cells infected with Δ123, but also revealed that this effect was also visible in B-cells infected with Δ3, 2/2*DSM and 3SM. The BHRF1 transcripts were also less abundant in all these samples, although the Δ3 mutant showed more drastic effects than the 2 seed mutants, thereby confirming the data gathered at the protein level.

The BHRF1 protein can be produced either from a lytic or from a latent promoter. To determine which of these forms is produced at an early stage of infection we infected B-cells with a virus that lacks the BZLF1 gene (ΔZ) that encodes a transactivator indispensable for the onset of lytic replication in B-cells (Fig 4f). This experiment showed that BHRF1 protein production is only slightly reduced at day 5 in B-cells infected with the ΔZ mutant, relative to wild type virus. Thus, it is the latent form of the BHRF1 protein that is predominantly produced at an early time point as previously suggested [11].

### The miR-BHRF1-3 miRNAs negatively regulate PTEN expression

**Fig 5:**
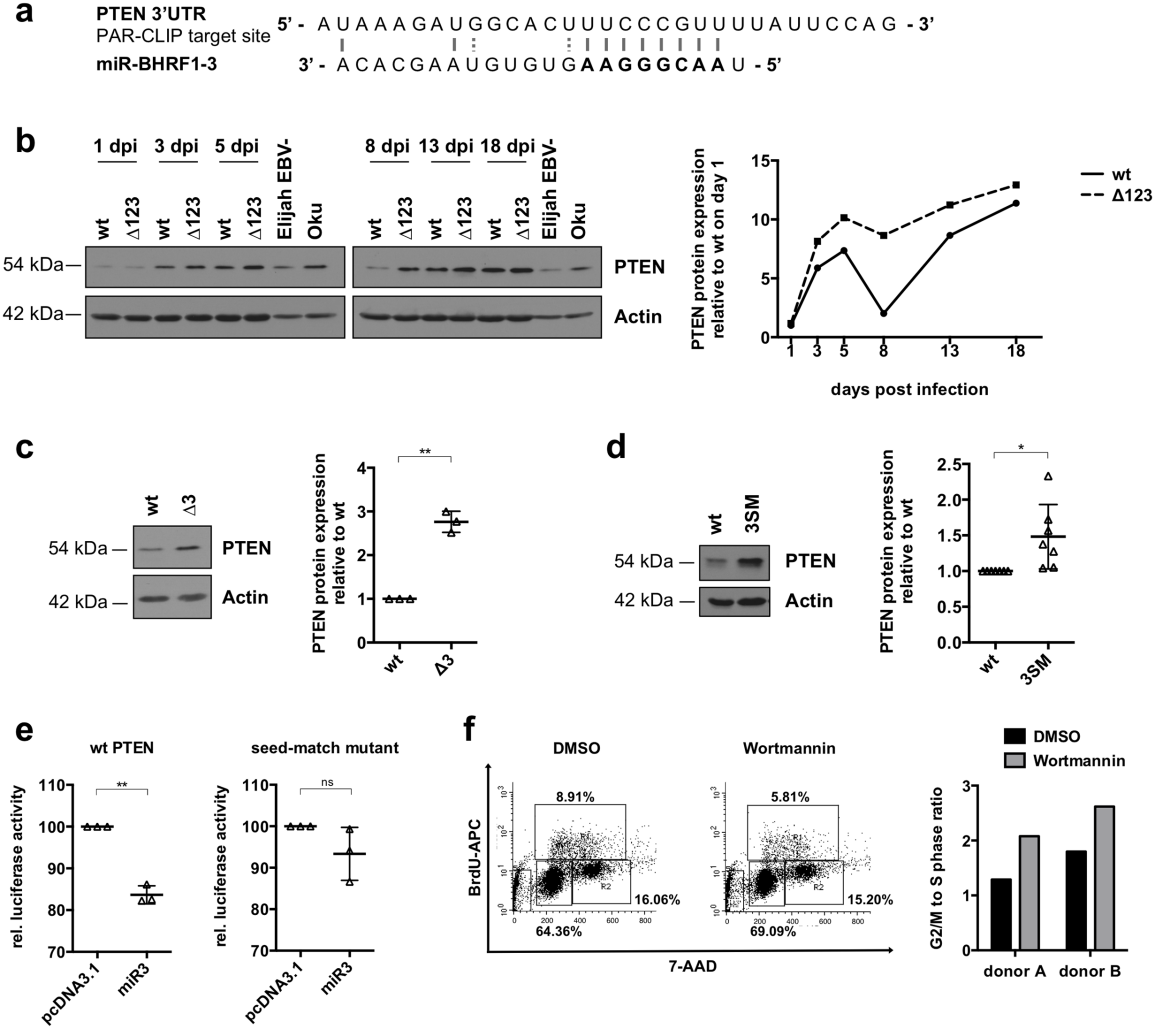
The BHRF1 miRNA negatively regulates PTEN expression. (a) The miR-BHRF1-3 target site in the PTEN 3’UTR according to a PAR-CLIP assay is shown (Skalsky et al.). (b) Primary B-cells were infected with B95-8 wild type (wt) or with the Δ123 recombinant and harvested at the indicated time points after infection for immunoblotting with a PTEN-specific antibody. Actin served as a loading control. Exactly the same amounts of Elijah and Oku-BL extracts were loaded on both gels to allow comparison of signal intensity between the two blots. The right panel shows the ImageJ-based quantification of the PTEN protein levels over time, normalized for differences in actin levels and signals obtained with Elijah and Oku-BL. PTEN expression in LCLs transformed by wt EBV, miR-BHRF1-3 (Δ3) single knockout (c), or 3SM (d). Actin served as a loading control. The right panels show the ImageJ-based quantification of PTEN expression for multiple blood samples. All values were normalized for differences in actin levels. (e) Luciferase assays performed with the PTEN 3’UTR or a seed-match mutant thereof by transfection of an expression plasmid that drives miR-BHRF1-3 expression or an empty pcDNA3.1 negative control. The results of three transfections are given relative to the luciferase activity signals recorded with the negative control. (f) 14 days old B-cells from two donors transformed with wild type EBV were treated for 24h with the PI3K inhibitor wortmannin (10nM) or the solvent DMSO. The cell cycle profile was determined by BrdU incorporation and quantified by FACS analysis. The results obtained with one B-cell primary sample are shown. This right panel depicts the ratio of cells present in the G2/M phase and in the S phase determined for two donors.

The data gathered so far confirmed that the B-cells infected with Δ123 have a reduction in cell cycle entry, even on feeder cells under conditions in which apoptosis is limited. We knew from previous work that a virus that lacks miR-BHRF1-3 displays similar, if less pronounced, abnormalities [6]. We looked for a gene implicated in the cell cycle control that would be regulated by miR-BHRF1-3. PTEN has previously been identified by a PAR-CLIP method as a potential target of miR-BHRF1-3 [17] (Fig 5a). Therefore, we tested expression of this protein at different time points after infection with Δ123 and wild type controls and found that it increased in intensity regularly from day 1 to day 5. After day 5 it became obvious that B-cells infected with the Δ123 virus expressed more PTEN than wild type counterparts (Fig 5b). We wished to confirm that this effect was due to the absence of miR-BHRF1-3 and assessed expression of PTEN in cells infected with the Δ3 or the 3SM virus. This experiment confirmed that cells infected with either of these single miRNA mutants evinced a stronger PTEN expression relative to wild type (Fig 5c and 5d). We also quantified PTEN expression in LCLs generated with ΔBHRF1 (S3 Fig). This assay could not reveal any difference in PTEN expression in these cells, relative to wild type controls.

We went on to perform a luciferase reporter assay in which the luciferase gene is fused with part of the 3’UTR of PTEN that contains the putative miR-BHRF1-3 binding site. We also included a negative control in which the putative miR-BHRF1-3 binding site had been mutated (seed-match mutant). Cotransfection of either of these constructs together with a miR-BHRF1-3 expression plasmid revealed a modest but statistically significant decrease in relative luciferase activity in the wild type PTEN 3’UTR fusion that was not visible in the seed-match mutant control (Fig 5e). This weak effect can at least in part be ascribed to the intrinsic low miR-BHRF1-3 expression level [6].

We then treated 2 14-days old EBV wild type-infected LCLs with wortmannin, an inhibitor of PI3K that mimics an activation of PTEN [18]. We found that the treatment of these cells with wortmannin decreased entry in S phase by one third and increased the G2/M to S phase ratio to a value close to the one we observed in the LCLs infected by Δ123 (Fig 5f). Thus, wortmannin treatment reproduced the cell cycle abnormalities observed after excision of the BHRF1 miRNAs. This supports the idea that the relative excess of PTEN seen in LCLs generated with Δ123 is responsible, partly or entirely for the observed abnormalities in cell cycle entry.

### miR-BHRF1-2 downregulates the synthesis of the BHRF1 protein in established LCLs

**Fig 6:**
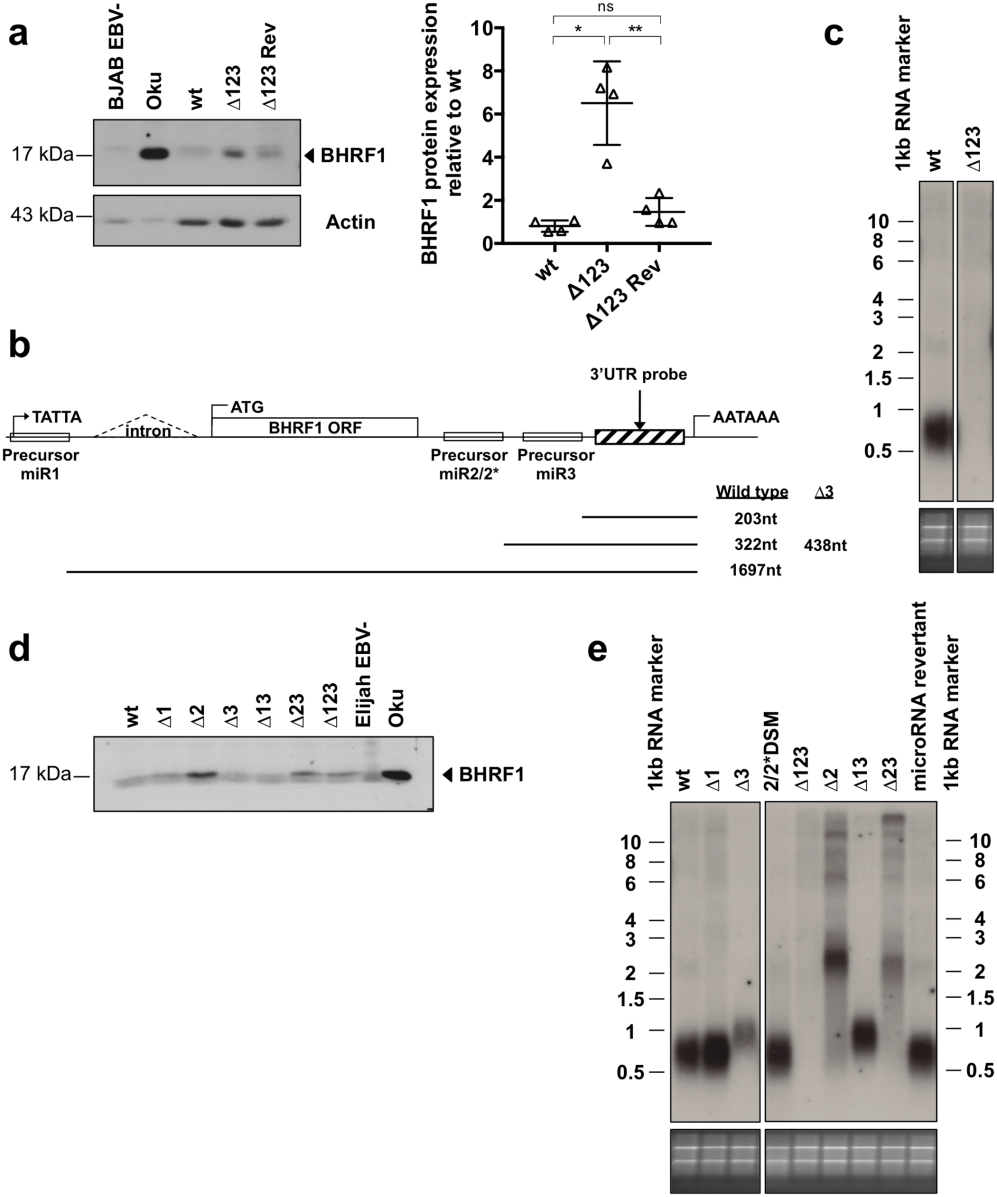
miR-BHRF1-2 downregulates the BHRF1 protein in established LCLs. (a) Western blot with a BHRF1-specific antibody performed on LCLs established from a primary B-cell sample infected with B95-8 wt, Δ123 or the Δ123 revertant virus (Δ123 Rev) virus and tested 2 months after infection. Actin served as a loading control. The EBV negative BJAB BL and the Wp-restricted Oku-BL served as negative and positive control for BHRF1 protein expression, respectively. The right panel shows the ImageJ-quantified BHRF1 protein expression in four donors 2 months after infection, normalized for differences in actin expression. (b) This schematic describes the BHRF1 locus and gives the length of the different BHRF1 RNA species that would arise from Drosha-mediated processing of the different miRNAs. The sizes are given exclusive of the polyA tails. (c) 1.5μg polyA+ RNA extracted from wild type- or Δ123-transformed LCLs was analyzed by northern blotting 45 days after infection. The blot was hybridized with a probe specific for the 3’UTR of BHRF1 downstream of miR-BHRF1-3, shown in (c). The bottom panel shows the agarose gel stained with ethidium bromide. (d) BHRF1 protein expression in LCLs 45 days after infection with a panel of viruses lacking one or several BHRF1 miRNAs was determined by western blotting. (e) 1.5μg polyA+ RNA was isolated from LCLs generated from one primary B-cell sample by infection with B95-8 wild type or the different single and double miR-BHRF1 knockout viruses. The northern blot was hybridized with a probe specific to the 3’UTR of BHRF1 downstream of miR-BHRF1-3, as depicted in (c).

We then turned our attention to infected cells that survived the critical day 5 to day 20 period and measured the expression of BHRF1 in cells established for more than 20 days. We found that, in accord with previous observations [11], established cell lines generated with wild type controls, hardly express BHRF1 (Fig 6a). In contrast, LCLs generated with Δ123 showed a clear expression of the protein. We repeated this experiment for 3 additional B-cell donors and obtained similar results that are given in Fig 6a. These results suggested that the BHRF1 mRNA that is used for translation of the protein could also be used as a template for miRNA processing. Therefore, we performed a northern blot on polyadenylated RNAs with a probe specific for the 3’ end of the BHRF1 gene (Fig 6b). This assay showed that the 0.5 kb transcript, that can only be generated by processing of the polyadenylated BHRF1 mRNA transcript at miR-BHRF1-2 or miR-BHRF1-3 (Fig 6c), was present in B-cells infected with wild type EBV but not in those infected with Δ123, confirming that the BHRF1 mRNA is cut by miRNA processing. To find out which of the BHRF1 miRNAs is responsible for this process, we infected B-cells with the Δ1, Δ2 and Δ3 mutants as well as with double mutants and measured BHRF1 expression in these LCLs. We found that only the LCLs infected by a virus that lacks miR-BHRF1-2 expressed higher levels of BHRF1 protein, demonstrating that processing of miR-BHRF1-2 cuts the potentially translated BHRF1 mRNA (Fig 6d). We then performed a northern blot with the same cells and could confirm that the BHRF1 3’UTR is not cut in the absence of miR-BHRF1-2. Thus, this miRNA plays an important role in the control of BHRF1 protein expression (Fig 6e). In the absence of miR-BHRF1-3, the 3’UTR was cleaved at miR-BHRF1-2. This resulted in a slightly larger signal in the northern blot. Thus, miR-BHRF1-2 and miR-BHRF1-3 are sequentially processed resulting in a cleavage of the primary BHRF1 transcript. This is consistent with our previous observation that efficient miR-BHRF1-3 processing requires the presence of miR-BHRF1-2 [6].

The set of BHRF1 miRNA mutants gave us the opportunity to investigate the mechanisms used by the virus to express the BHRF1 protein and more generally the transcription of the BHRF1 locus in more detail. To this end, we used again northern blots to assess the nature of the transcripts that entail the BHRF1 gene with probes specific to the BHRF1 open reading frame, its intron, or to the origin of lytic replication (oriLyt) which is located directly 5’ of the BHRF1 locus (S4 Fig). This analysis led us to propose a model in which large unspliced polyadenylated transcripts encompassing the oriLyt region and the BHRF1 ORF are a source of BHRF1 protein. These transcripts also yield BHRF1 miRNAs, whose processing give rise to smaller and smaller RNA products.

Hybridization of total RNA from wild type-infected LCLs with the BHRF1 ORF probe revealed a prominent 1.3 kb transcript that contains the BHRF1 intron but neither oriLyt not the BHRF1 3’UTR. It also evidenced the existence of larger transcripts of very faint intensity, some of which are larger than 8 kb. This pattern reproduces what we previously observed with other wild type LCLs at 60 days and suggests that the difference in the pattern of BHRF1 transcripts observed at day 5 post-infection might be related to the fact that BHRF1 miRNAs are not fully expressed at that time, or at least not proportionally to total BHRF1 transcription (Fig 2b). Indeed, in mutants that lack miR-BHRF1-1 or miR-BHRF1-2, the 1.3 kb transcript shifted to transcripts that were larger than 8 kb in size and of variable intensity, depending on the mutants. In mutants that lack miR-BHRF1-2, there was in addition a band at approximately 2.3 kb. Importantly, miR-BHRF1-3 is not required for the generation of the 1.3 kb transcript. Thus, the 1.3 kb transcript is generated from larger fragments through the processing of miR-BHRF1-1 and miR-BHRF1-2, entails the BHRF1 intron but not oriLyt, and has the size of the BHRF1 RNA fragment between these 2 miRNAs. In all probability, it is identical to the transcript generated by miRNA processing that was previously identified [19, 20]. This 1.3 kb transcript was already visible at day 5, but, in contrast to what we saw in established cell lines, larger signals of equal or even stronger intensity were also present (Fig 2b). However, it is important to note that the total BHRF1 transcription at 1.5 to 2 months post-infection is much reduced compared to the first days post-infection, suggesting that processing of the BHRF1 miRNAs leads to a disappearance of high molecular weight BHRF1 RNAs, except if the BHRF1 transcripts are in massive excess.

The investigation of poly A+ mRNAs revealed that LCLs produced additional RNAs. LCLs infected with wild type controls contain one clear signal at 2.2 kb as well as larger, much fainter signals. The 2.2 kb fragment contains the BHRF1 intron, the BHRF1 ORF but not the oriLyt fragment. It is absent in LCLs infected with mutants that lack miR-BHRF1-1 or -3 but is very abundant and slightly larger in cells infected with mutants that lack miR-BHRF1-2. The LCLs infected by viruses that lacked miR-BHRF1-1 or -2, including the Δ123 mutant, also displayed an accumulation of transcripts ranging from 4 to larger than 10 kb, in line with the assumption that large BHRF1 transcripts serve as a source of these miRNAs. The dominant role of miR-BHRF1-2 processing was also visible in the LCLs generated with Δ13 in that they also produced the large transcripts, but at a lower expression level than those infected with viruses that lack this miRNA. These large transcripts contain the BHRF1 intron, as well as the oriLyt sequence. We conclude that the 2.2kb transcript is generated from large transcripts that encompass oriLyt, contains the BHRF1 intron and open reading frame and the BHRF1 3’UTR, requires the presence of miR-BHRF1-1 for its generation and accumulates in the absence of miR-BHRF1-2. Taking into account that the distance between miR-BHRF1-1 and the end of the BHRF1 polyA tail is 2.2 kb, we conclude that the 2.2 kb RNA represents a polyadenylated transcript that begins at miR-BHRF1-1 and runs through to the BHRF1 polyA. It is important to note that this transcript runs slightly higher in the LCLs infected by Δ23 and even higher in those infected with Δ2. This fits with the fact that the BHRF1 transcript is 46 and 126 ribonucleotides longer in the Δ23 and the Δ2 mutants, respectively, than in the wild type BHRF1 gene. Altogether, we conclude that the 2.2 kb polyadenylated transcript is very likely to represent a precursor form of the 1.3 kb transcript identified in the total RNA blot that is itself generated upon miR-BHRF1-2’s processing. The abundance of the larger BHRF1 transcripts was inversely related to the existence of the 2.2 kb transcripts and their existence was strongly dependent on the absence of miR-BHRF1-2. In the absence of miR-BHRF1-1, there were also some larger but less strongly expressed high molecular weight BHRF1 transcripts. This points to the central role of miR-BHRF1-2 in the processing of these large transcripts. We noticed that there is an inverse correlation between the expression of the BHRF1 protein and the presence of shorter BHRF1 RNA forms. This suggests that the large polyadenylated mRNAs that encompass BHRF1 are the source of the BHRF1 protein and that they are destroyed during BHRF1 miRNA processing, in particular though processing of miR-BHRF1-2. LCLs generated with Δ3 express high levels of miR-BHRF1-2 [6] and this fits with the observation that this cell line does not express any of the BHRF1 intermediates but only their final product, the 1.3 kb RNA fragment.

### LCLs established with Δ123 are more resistant to apoptosis

**Fig 7:**
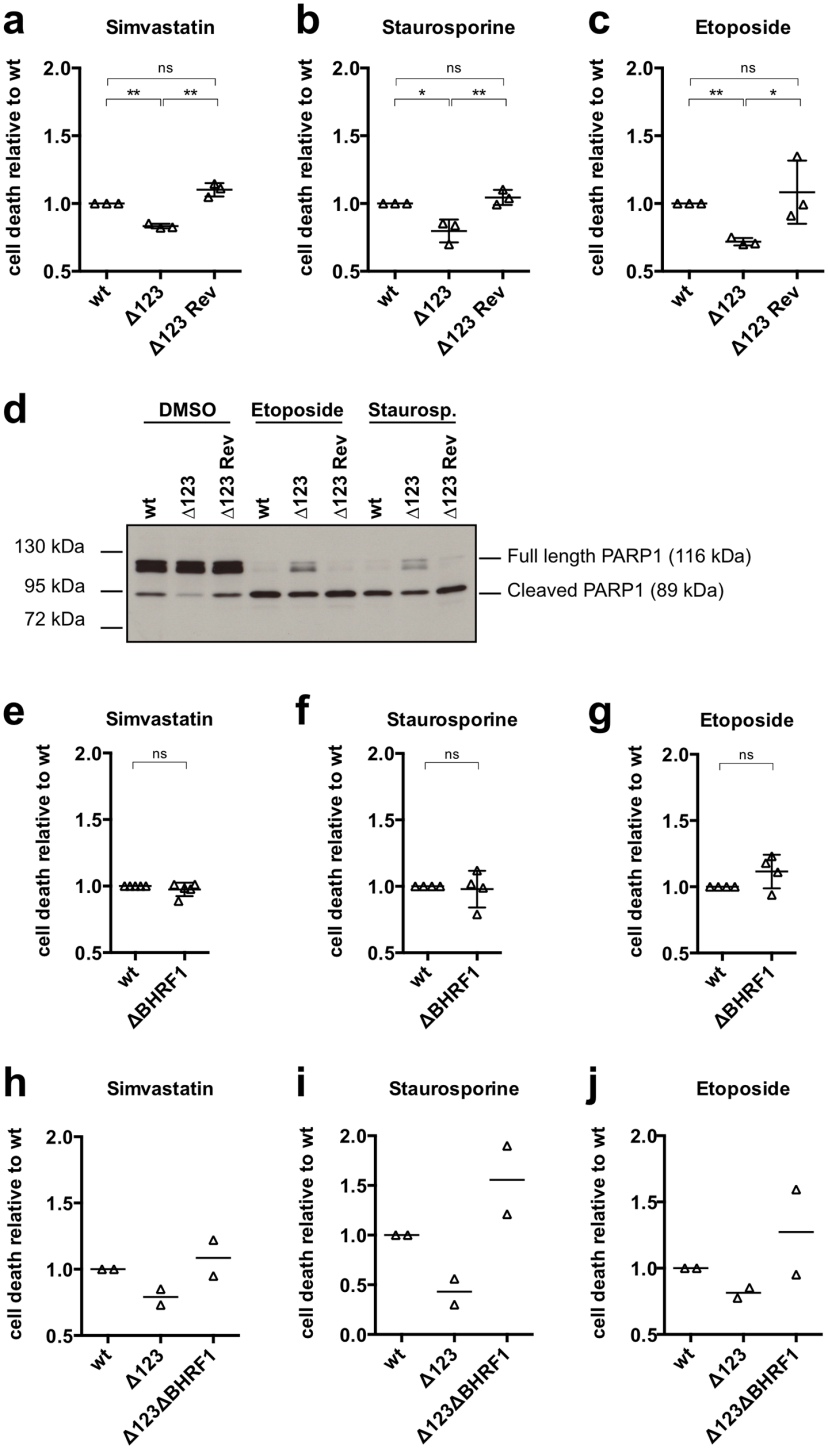
LCLs established with Δ123 are more resistant to apoptosis. Established, 2–3 months old LCLs transformed with wild type, Δ123, or the Δ123 revertant were treated with different apoptosis-inducing agents or the respective solvent control. Cell samples were stained with Annexin-V and 7AAD and the number of apoptotic cells was determined by FACS and normalized for the percentage of dying cells in the respective solvent-treated control samples. Three LCLs established from different buffy coats were tested for each experiment. The cell death rate of the tested samples is given as its ratio with the death rate of samples infected with the wild type virus. (a) LCLs were treated 5 days with simvastatin (2μM) or EtOH as solvent control. (b) LCLs were treated with staurosporine (4μg/ml) or DMSO as solvent control for 20hrs. (c) LCLs were treated with etoposide (4μg/ml) or DMSO as solvent control for 20 hrs. (d) PARP western blot of one triplet of LCLs is shown after 20 hrs of treatment with etoposide, staurosporine or DMSO as solvent control. The same experiment was repeated for LCLs established from 4 different buffy coats generated by infection with wild type, Δ123, ΔBHRF1 (e, f, g) or from 2 additional samples infected with the wild type or Δ123ΔBHRF1 (h, i, j). Cell death rates were assessed after treatment with simvastatin (e and h), staurosporine (f and i), or etoposide (g and j).

We then went on to assess the functional consequences of BHRF1 overexpression. To this end, we provoked apoptosis in LCLs established with B-cells from multiple donors and generated by infection with Δ123 or with wild type controls. This was achieved by incubating the cells with etoposide, staurosporine or simvastatin for a variable length of time and staining these cells with Annexin-V and 7AAD (Fig 7a, 7b and 7c). Treatment with these drugs gave rise to massive cell death in all samples, although the killing efficiency was significantly higher in the wild type LCLs than in LCLs generated with Δ123. We confirmed these data with a PARP cleavage assay (Fig 7d). Whilst some cells infected with Δ123 retained some intact PARP after treatment with etoposide or staurosporine, this was not the case for cells infected with wild type viruses. We then performed similar experiments with B-cells infected with ΔBHRF1 and Δ123ΔBHRF1. We found that LCLs generated with ΔBHRF1 or with wild type viruses do not significantly differ in their response to apoptotic stimuli (Fig 7e, 7f and 7g). This fits with the observation that BHRF1 is expressed at very low levels in established LCLs [11]. Fig 7h, 7i and 7j show that B-cells infected with Δ123ΔBHRF1were much more sensitive to provoked apoptosis than Δ123 LCLs, and even showed more cell death upon induction than the wild type controls. This suggests that in the absence of the BHRF1 protein, infected B-cells rely on the BHRF1 miRNAs to withstand apoptotic stimuli. These results concur with our observation that at an early time point after infection, B-cells infected with a virus such as Δ123 that expresses low BHRF1 protein levels are crucially dependent on this residual activity as seen by infection with the Δ123ΔBHRF1 mutant (Fig 3).

### p27 expression in B-cells infected with Δ123

**Fig 8:**
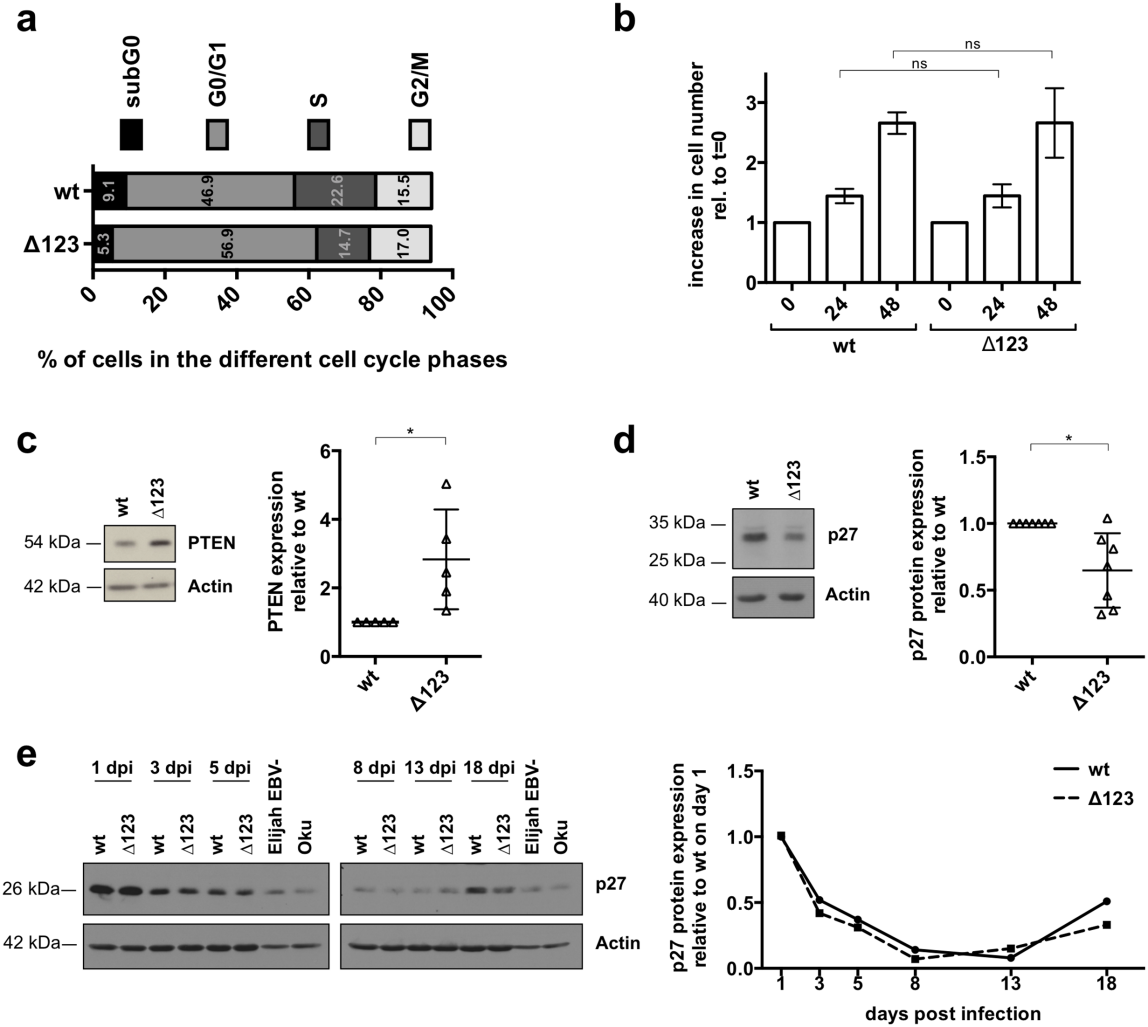
p27 expression in B-cells infected with Δ123. (a) We performed a BrdU incorporation assay with LCLs transformed by wild type or Δ123 viruses. The figure shows the results of the FACS analysis two months after infection. (b) 2 months-old cells infected with wild type or the Δ123 virus were brought to a concentration of 3x10^5^ cells per ml and the increase in cell numbers was determined by counting trypan blue-negative cells 24 and 48 hours thereafter. The growth rate is given relative to initial cell numbers. The growth kinetics of four donors were determined twice in independent experiments. (c) Western blot for PTEN expression from one representative donor infected with B95-8 wt or B95-8 Δ123. LCLs were analyzed 2.5 months after their establishment. Actin served as a loading control. ImageJ quantified PTEN expression in three donors 2.5 month after infection is shown in the right panel. All values were normalized for differences in actin expression. (d) We measured p27 expression in seven 2–3 months old LCLs established with wild type EBV or with Δ123 using a western blot analysis. The left panel shows one example; the right panel gives the results of ImageJ-based quantification for all tested donors. The individual values were normalized for differences in actin levels and p27 levels are depicted relative to the respective wild type control. (e) p27 expression was monitored between day 1 and 18 after infection of primary B-cells with wild type or Δ123 viruses. Actin served as a loading control. The right panel depicts the ImageJ-based quantification of the p27 protein levels normalized for differences in actin expression.

We assessed the cell cycle characteristics of LCLs that had been established for more than 35 days using a BrdU incorporation assay (Fig 8a). Although the percentage of cells in S phase remained lower in LCLs infected by Δ123 relative to those generated with wild type viruses, the ratio between these numbers increased from 0.42 (Fig 1c 5.7/13.5) to 0.65 (Fig 8a 14.7/22.6). We also generated growth curves with LCLs transformed by Δ123 and wild type virus and found that both types of LCLs grow at very similar rates (Fig 8b). However, as seen in Fig 8c, PTEN levels remained higher in cells infected with the Δ123 mutant relative to wild type. Therefore, LCLs infected with Δ123 must have acquired additional changes in their cell cycle regulation after several weeks in culture. We screened the expression of key regulators of the cell cycle, including p53, Rb and the CDKN family in the different types of LCLs by western blot. This analysis revealed that CDKN1/p27 is frequently expressed at lower levels in established LCLs infected by the Δ123 mutant relative to wild type controls after more than one month in culture (Fig 8d). We monitored expression of p27 during the day 5 to day 18 crucial period and found that the protein levels of p27 decreased rapidly after infection to nearly disappear at day 13 p.i. However, this expression re-increased after day 18, albeit less strongly in B-cells infected with the Δ123 mutant (Fig 8e). These data argue against a direct impact of the BHRF1 miRNAs on p27 and establish a parallel between the decrease in p27 and the recovery of the cell growth rate in B-cells infected with Δ123.

## Discussion

The clarification of the molecular mechanisms fine tuned by miRNAs is frequently a difficult undertaking and the BHRF1 miRNAs are no exception to that rule. CLIP-based strategies have identified potential targets of these miRNAs but the identity of the crucial proteins that they modulate remains enshrouded in mystery [17]. We have evaluated in detail the role served by the BHRF1 miRNAs during the first weeks of EBV-mediated B-cell transformation and found that cells infected with a virus that lacks the three BHRF1 miRNAs undergo on average twice as much apoptosis than cells infected with wild type controls between day 5 and day 20. Our results are only partially concordant with an earlier report that described a massive apoptosis in cells infected with a virus devoid of the BHRF1 miRNAs during the first five days of infection, followed by a quick recovery of cell numbers 10 days after infection [4]. These data are difficult to reconcile with the fact that the BHRF1 miRNAs are fully produced only 5 to 8 days after the onset of infection [21, 22] and we indeed did not see any differences between cells infected with the Δ123 mutant and cells infected with wild type controls during the first 5 days of infection. One difference between the studies lies in that we investigated the infected B-cells at the single cell level and used a larger panel of markers to characterize apoptotic cells. Indeed, positive staining for Annexin-V used in the study by Seto et al. is not specific for apoptosis and is found in many other modes of cell death [23].

We found that the expression of the viral bcl-2 homolog BHRF1 is modulated by the BHRF1 miRNAs in an unexpectedly complex manner. During the first days of infection, the BHRF1 protein has previously been reported to be expressed at relatively high levels and we could confirm this observation [11]. However, B-cells infected with the Δ123 virus hardly express BHRF1, suggesting that the observed increased apoptosis after infection with Δ123 is due to a reduction in BHRF1 expression. Indeed, a virus that lacks the BHRF1 protein and a virus that lacks the BHRF1 miRNAs induce similar phenotypes in infected B-cells. Interestingly, cells infected with ΔBHRF1 not only exhibited an increased apoptotic rate but also a reduced entry into cell cycle. At the present stage of the work, it is unclear whether the cell cycle abnormalities are a consequence of increased apoptosis, or whether this reflects a specific so far unrecognized, PTEN-independent, property of the BHRF1 protein. Our results are discordant with those obtained by Altmann et al. who found that only a virus lacking both BHRF1 and BALF1 has a reduced transforming ability as a consequence of enhanced apoptosis upon infection of B-cells [12]. However, in this study, the authors showed growth curves of B-cells infected with a virus that lacks the BHRF1 protein that are reminiscent of our own results and clearly differ from those obtained with wild type virus. Moreover, these authors also discussed the possibility that a virus that lacks BHRF1 only might also induce an abnormal phenotype without reaching definite conclusions.

Importantly, B-cells infected with a virus that lacks both the BHRF1 miRNAs and the BHRF1 protein undergo a strong degree of apoptosis starting at day 5 post-infection and increasing until day 20 at which time infected cells die. Thus, the phenotype induced by Δ123ΔBHRF1 is much more severe than the one induced by Δ123. Importantly, BHRF1 is expressed, although at low levels, shortly after infection with Δ123. This suggests that the very low amounts of BHRF1 are sufficient to limit the apoptosis in cells infected by Δ123. However, it is important to note that the construction of the Δ123ΔBHRF1 mutant led to a complete deletion of this gene. Therefore, although it is unlikely, we cannot exclude that so far unidentified genetic elements might be altered in this mutant. Moreover, we found that the low BHRF1 expression level in established LCLs infected with wild type EBV has no significant impact on drug-induced apoptosis, as a LCL infected with a virus that lacks the BHRF1 protein but expresses the BHRF1 miRNAs is not more sensitive to provoked apoptosis than wild type controls. This underlines again the role of the BHRF1 miRNAs in the regulation of the apoptotic status in the absence of the BHRF1 protein.

The observation that the phenotype induced by Δ123ΔBHRF1 is more severe than the one observed in B-cells infected with ΔBHRF1 also indicates that the function of the BHRF1 miRNAs is not subsumed by the modulation on BHRF1 protein levels. Therefore, we looked for additional targets of the BHRF1 miRNAs and the recognition that B-cells infected by Δ123 display a reduced entry in cell cycle oriented the search. We also knew that a recombinant virus lacking miR-BHRF1-3 displays some cell cycle abnormalities and therefore looked for potential miR-BHRF1-3 targets identified in the PAR-CLIP assays [6, 17]. This led to the identification of PTEN as a protein whose expression is downregulated by miR-BHRF1-3. This effect was visible as early as day 5 post-infection with Δ123 and persisted in established LCLs. Treatment of wild type LCLs with the PI3K inhibitor wortmannin gave rise to a decrease in cell cycle entry, reproducing the cell cycle alterations observed in B-cells infected with Δ123 and suggesting that the PI3K pathway is active in EBV-transformed B-cells, as previously suggested [24]. Brennan et al. found that inhibition of PI3K leads to a downregulation of cyclin D2 and cyclin D3, combined with an increase in p27 that causes growth arrest [24]. PTEN is an inhibitor of PI3K, and its increased expression in LCLs is likely to reduce the PI3K activity in these cells. Therefore, downregulation of PTEN through miR-BHRF1-3 is expected to facilitate cell division in LCLs generated with wild type EBV. PTEN is also targeted by cellular miRNAs such as the miR-17~92 cluster, that is expressed in LCLs [17, 25, 26]. Thus, viral and cellular miRNAs seem to collaborate to downregulate its expression. Moreover, PTEN, through its repression of the PI3K/Akt pathway, also negatively modulates the apoptotic status of the cell [27]. We found that miR-BHRF1-3 is able to weakly downregulate a luciferase-PTEN 3’UTR reporter gene. However, the difficulties in expressing miR-BHRF1-3 render interpretation of these results difficult. Altogether, these results, in combination with those of the PAR-CLIP assay suggest that miR-BHRF1-3 directly targets PTEN, but other mechanisms cannot be excluded.

We found that the BHRF1 miRNAs influence the dynamic expression profile of Wp-driven and BHRF1-specific transcripts. In their absence, the peak of transcription shortly after infection was delayed by about one week, after which transcription remained higher than in the wild type counterparts. We could show that BHRF1 protein expression depends on miR-BHRF1-3 and miR-BHRF1-2 seed regions and identification of their targets should shed light on their exact mechanism of action.

After 30 days, the infected cells recovered a normal growth rate, as assessed by growth curves and PH3 staining. The cell cycle profile of B-cells infected with Δ123 substantially improved at this point, with an increased proportion of cells that enter the S phase, although it did not reach wild type levels. These LCLs were also more resistant to drug-induced apoptosis.

Accordingly, the BHRF1 protein expression profile differed between established LCLs and freshly infected B-cells. LCLs established with Δ123 expressed more BHRF1 protein than their wild type counterparts and this explains the increased resistance to apoptosis, as LCLs infected with Δ123ΔBHRF1 or ΔBHRF1 have lost this property. We could identify miR-BHRF1-2 as the miRNA that is mainly responsible for the downregulation of the BHRF1 protein after establishment of the LCLs. The sequential processing of miR-BHRF1-2 and miR-BHRF1-3 that results in the cleavage of the 3’ end of the BHRF1 mRNA suggests that miR-BHRF1-2 does not solely act through the classical RISC-mediated target mRNA regulation, but highlights another mechanism through which a miRNA regulates protein expression. Therefore, we have shown that the latent BHRF1 mRNAs can act as a template for BHRF1 translation but can also be used by the Microprocessor machinery to generate the BHRF1 miRNAs and that both processes are in competition for access to the mRNA. The alternative use between protein translation and miRNA processing was previously reported for the cellular gene follistatin and miR-198 that are coded on the same mRNA [28]. Northern blot analyses have shown that infected B- cells produce abundant probably Wp-initiated high molecular weight mRNAs that contain the BHRF1 ORF. The exact identity of the BHRF1 transcripts that are translated remains unknown but we know that they contain the BHRF1 intron. Furthermore, analysis of established LCLs has also shown that high molecular weight BHRF1 RNAs are polyadenylated and probably also contain oriLyt-specific sequences. BHRF1 transcripts initiated at oriLyt have been previously identified and could potentially be the source of the protein [29–31]. Moreover, large polyadenylated RNAs containing intronic BamHI W sequences linked to the EBNA-LP, EBNA2, and BHRF1 polyA sequences have been also detected in EBV-infected cells [29].

The observation that the same miRNAs can successively activate and repress BHRF1 protein expression is puzzling but we could identify clear differences between these 2 stages. The BHRF1 transcription rate is much higher at an early time point than after establishment of the LCL. At this time, Wp-driven transcription dominates and it has previously been found that this promoter drives the expression of BHRF1 [11]. Furthermore, we also found that whilst the stimulatory effects of miR-BHRF1-2 and -3 depended on their seed regions, the repressive effects of the BHRF1 cluster was mainly mediated by miR-BHRF1-2 processing. We hypothesize that, at the beginning of infection the strong Wp-driven BHRF1 transcription, boosted by miR-BHRF1-2 and -3 favors BHRF1 translation. This would imply that high transcription rates disadvantages miRNA processing or that Wp-driven transcripts are less accessible to the Microprocessor machinery. At this stage, the ratio between transcripts processed or not by the Microprocessor machinery allows BHRF1 protein synthesis. When Wp-mediated transcription ceases, BHRF1 transcription is reduced, the BHRF1 miRNAs are efficiently processed and the BHRF1 translation is reduced to a minimum. Why would infected cells first express BHRF1 and then downregulate its expression? Our data show that BHRF1 expression protects infected B-cells against apoptosis at the beginning of the infection. The kinetic of expression of BHRF1 and of the viral latent membrane protein 1 (LMP1), two proteins endowed with anti-apoptotic properties [9, 32], is strikingly opposite. LMP1 is expressed at low levels shortly after infection and reaches its plateau of expression only 21 days after infection, which is exactly the period at which the increased apoptotic rate in cells infected with Δ123 normalizes [33]. Thus, it is possible that BHRF1 assumes LMP1 anti-apoptotic functions until this protein reaches its optimal expression level. However, virus-infected cells need to protect themselves from the immune response. As BHRF1 is efficiently targeted by the T cell response, its expression needs to be downregulated to a minimum when it is not anymore needed [34].

In contrast to BHRF1, PTEN levels remained high in cells infected by Δ123 and this is likely to explain the persisting reduced entry in S phase in these cells. This fits with the concept that this protein is targeted by miR-BHRF1-3, whose levels reach a plateau after day 5 to 8 in infected cells [21, 22]. Nevertheless, the improvement in S phase entry in established LCLs prompted us to screen the expression of cell cycle regulators in these cells and we identified p27 as a protein whose expression is reduced after 30 days in culture. The expression of p27 varies markedly over time in LCLs and follows a complex pattern. As expected for non-dividing cells, the expression of this protein was high one day after infection. It decreased then to become hardly visible 2 weeks after infection. However, it then started to increase again but remained lower from this stage in cells infected with Δ123 viruses.

The reduced expression of p27 suggests that infected cells counteract the effects of PTEN overexpression. Indeed, PTEN blocks the repressive effects of Akt on p27 and thus increases p27 expression [27]. At this stage, we cannot distinguish between a selection process that facilitates the growth of cells with low p27 in the context of increased PTEN expression and an indirect effect of the BHRF1 miRNAs. We favor the first possibility because the expression of p27 re-increases in wild type LCLs, even in the presence of the BHRF1 miRNAs and thus seems to be independent of them.

In conclusion, we have identified BHRF1, PTEN and p27 as direct or indirect targets of the BHRF1 miRNA cluster. These 3 proteins regulate the apoptotic status and entry into S phase, two essential cell functions. Although it is likely that other important targets of the BHRF1 miRNAs remain to be discovered, many of the phenotypic traits evinced by the Δ123 can be explained by the modulation of these 3 proteins. The expression of the BHRF1 miRNAs increases BHRF1 protein production and reduces PTEN production after 5 days post-infection to facilitate cell division. At a later time point, the BHRF1 miRNAs reduce expression of the BHRF1 anti-apoptotic protein and indirectly increase expression of p27, two events associated with a reduced propensity to neoplastic cell transformation [35, 36] that is beneficial for long-term persistence in the host. BHRF1 has gained increasing attention in recent years for its important function in LCLs and in Burkitt’s lymphomas. BHRF1 also represents a potentially very attractive therapeutic target [37]. It is therefore not surprising that its expression is tightly modulated within the infected cell. It is interesting to note that the appearance of a miRNA cluster within the BHRF1 locus is relatively new during evolution and is restricted to gammaherpesviruses with a tropism for B-cells and may be related to the need to restrict efficient MHC class I and class II presentation from B-cells [34, 38, 39].

## Materials and Methods

### Cells

Cells were kept in RPMI-1640 (Life Technologies) supplemented with 10% FBS (Sigma) at 37°C in a 5% CO_2_-buffered humid atmosphere. Primary B-cells infected with EBV were kept in RPMI/20%FBS until establishment of the cell line and 100μg/ml Hygromycin B (Calbiochem) was added to HEK 293 producer cells to induce selection pressure in cells stably carrying the EBV-BAC. HEK 293 cells are neuro-endocrine cells obtained by transformation of embryonic epithelial kidney cells with adenovirus (ATCC: CRL-1573). Primary B-cells were isolated from adult human blood buffy coats by Ficoll (GE healthcare) density gradient centrifugation and the CD19+ B-cell population was purified using CD19 PanB Dynabeads (Life technologies) and DETACHaBEAD CD19 (Life technologies). Elijah-5E5 is an EBV-negative subclone of the EBV positive Burkitt’s lymphoma cell line Elijah (kindly provided by A.B. Rickinson). BJAB is an EBV-negative Burkitt’s lymphoma cell line (kindly provided by A.B. Rickinson) [40]. Oku-BL is a Wp-restricted Burkitt’s lymphoma cell line (kindly provided by A.B. Rickinson) [10]. WI38 are primary human lung fibroblasts (ATCC: CCL-75 [41]).

### Ethics statement

All human primary B-cells used in this study were isolated from anonymous buffy coats purchased from the Blood Bank of the University of Heidelberg. No ethical approval is required.

### EBV recombinants

The recombinant EBV wild type BAC (B95-8 wt/2089) was constructed by introducing the bacterial F-factor, GFP, a chloramphenicol resistance gene and a hygromycin selection marker in the EBV strain B95-8 [42]. The construction of the miR-BHRF1 deletion mutant and revertant [5], of the miR-BHRF1 single mutants [6] and of the BZLF1 deficient virus ΔZ [43] were reported previously. The Δ123ΔBHRF1 mutant was obtained by exchanging the complete BHRF1 locus of the B95-8 wild type BAC (EBV coordinates 53758:55278 (V01555.2)) with a kanamycin resistance cassette by homologous recombination [44]. This cassette was amplified from pCP15 using the primers 1422 (ATGTGGGGGT GGAAATATGA GCAAGAATAA GGACGGCTCC AACAGCTATG ACCATGATTA CGCC) and 683 (ATTTTAACGA AGAGCGTGAA GCACCGCTTG CAAATTACGT CCAGTCACGA CGTTGTAAAA CGAC). We used En Passant Mutagenesis [45] to construct a BHRF1 deficient recombinant virus (ΔBHRF1) in which the BHRF1 ATG start codon (54376:54378 (V01555.2)) was replaced by ATTAG in GS1783, a bacterial strain that contains the B95-8 wild type BAC. To amplify the kanamycin resistance gene from the pepKanS plasmid, the primers 1855 (CCTCTTAATT ACATTTGTGC CAGATCTTGT AGAGCAAG*AT TA*AGTAGGGAT AACAGGGTAA TCG) and 1856 (TATACACAGG GCTAACAGTA TCTCCCTTGT TGAATAGGC*C TA*ATCTTGCT CTACAAGATC TGGCACAAAT GTAATGCCAG TGTTACAACC AATTAACC) were used.

To generate the 3SM seed mutated recombinant virus, three point mutations were introduced in the seed region of miR-BHRF1-3 (AACGGGA converted to AACGTTG). To this end, the nucleotides in the mature seed as well as the complementary miR-BHRF1-3* strand (55261:55263 and 55307:55309 (V01555.2)) were mutated using En Passant mutagenesis. The kanamycin resistance gene from the pepKanS plasmid was amplified using the primers 2125 (CAATTGGGTG TCCTAGGTGG GATATACGCC TGTGGTGTTC TAACGTTGAG TGTGTAAGCA CACACGTAAT TTGCAAGCGG ATAAGTAGGG ATAACAGGGT AATCG) and 2126 (CTCAGTTATT TCTTTAGTAT CTTGTCCTTG TGTTATTTTA ACGCCAAGCG TGAAGCACCG CTTGCAAATT ACGTGTGTGC TTACACACTC AACGTTAGCC AGTGTTACAA CCAATTAACC) and introduced into B95-8 Δ3 in GS1783.

Successful construction of all clones was verified by sequencing of the mutated region and integrity of the complete genome was confirmed by restriction enzyme digestion of the BAC clones and of the stably transfected HEK 293 producer cells.

Induction of HEK 293 producer cells to generate virus supernatants for B-cell infection and the quantification of virus titers were performed as previously described [5].

### B-cell infections

10^6^ primary B-cells were infected using a MOI of 10 EBV genome equivalents per cell for 2 hrs at RT and cultured in RPMI/20%FBS. Four to five days after infection with EBV, B-cells initiate permanent growth that gives rise to the establishment of cell lines termed lymphoblastoid cell lines (LCL). For transformation assays, 5×10^2^ cells were infected with a MOI of 0.01, that is enough viruses to induce GFP expression in 1% of infected cells [5], for 2 hrs at RT and plated on 96-well plates coated with 50Gy irradiated WI38 fibroblasts. 48 wells per donor and virus supernatant were seeded and the number of transformed wells was determined 30 days post-infection.

### Analysis of mitosis and the cell cycle profile

Cells were washed once in 1xPBS, spread on glass slides (Medco) and air-dried. For phospho-histone 3 staining (PH3; 1:100; Cell Signaling), cells were fixed in 4% PFA for 20 min at RT, washed 5 min in 1xPBS, permeabilized by immersion in 1xPBS/0.5% Triton-X for 2 min and washed again for 5 min in 1xPBS. Cells were incubated with the primary antibody diluted in PBS/10%HINGS (heat inactivated; Gibco) for 30 min at 37°C in a humid chamber and washed 3 times 5 min in 1xPBS. Secondary goat anti-rabbit Cy3 conjugated antibody (1:1200; Dianova) was applied for 30 min at 37°C in a humidity chamber, cells were washed 3 times for 5 min in 1xPBS and the DNA was counterstained using Hoechst 33258 for 2 min at RT. The cells were embedded in 90% glycerol and analyzed by fluorescence microscopy (Leica DM5000 B).

A triple staining of the mitotic spindle (α-tubulin), the centromeres (centrin-2) and the DNA (DAPI) was used on cells flattened by cytospin to analyze mitosis. To this end, cells were harvested, washed twice in 1xPBS/3% FBS and 5×10^4^ cells were spun on slides (Tharmac, Cytoträger) in 100μl PBS/3%FBS using the cytospin 4 (Thermo; EZ single cytofunnel, Thermo) for 10 min at 2000rpm, maximum acceleration. Cells were air-dried, fixed in PFA and permeabilized using PBS/Triton-X as described above. Unspecific protein binding was blocked for 45 min in PBS/3%BSA at room temperature in a humid chamber. Cells were stained with rabbit α-centrin-2 (1:100; Santa Cruz) and mouse α-α–tubulin (1:4000; Sigma) in PBS/3%BSA for 2 hrs at 37°C in a humidity chamber, washed 5 times in 1xPBS, followed by incubation for 2 hrs in the secondary antibodies diluted in PBS/3%BSA (goat α-mouse IgG-Alexa488, 1:300, Invitrogen; goat α-rabbit Cy3, 1:1200, Dianova). After washing 5 times in 1xPBS, the cells were embedded in ProLong Gold antifade reagent with DAPI (Life technologies) and analyzed at a magnification of 630x.

For BrdU incorporation assays, cells were adjusted to 5×10^5^ cells per ml one day prior to cell cycle analysis. 5×10^5^ cells were pulsed with 10μM BrdU for 35 min at 37°C and stained using the APC BrdU Flow Kit (BD Pharmingen) according to the manufacturers instructions. The cell cycle profile was determined with a FACSCalibur flow cytometer. This assay allowed recognition of cells in G1/G0 (BrdU negative, 7AAD single DNA content), S phase (BrdU positive, shifting from single to double DNA content (7AAD)) and the G2/M phase (BrdU negative, double DNA content (7AAD)).

The growth rate of established LCLs was determined using the trypan blue (Sigma) dye exclusion method. The number of viable cells was determined 24 hrs and 48 hrs after adjusting the cultures to a density of 3x10^5^ cells per ml.

### Apoptosis/viability assays

Cells were harvested at indicated time points, washed once in 1xPBS, spread on glass slides, fixed and stained for cleaved caspase 3 (Casp3; 1:400; Cell Signaling) as described above for PH3 and single cells were analyzed by fluorescence microscopy. For detection of apoptosis using the TUNEL technology (terminal deoxynucleotidyl transferase (TdT)-mediated dUTP nick end labeling), cells were washed once in 1xPBS, spread on glass slides (Medco), the DNA double strand nicks were enzymatically labeled with the In Situ Cell Death Detection Kit, TMR red (Roche) according to the manufacturers instructions and analyzed by fluorescence microscopy.

Following induction of apoptosis, cell death was determined using Annexin-V-Alexa647 (Roche). To this end, 10^6^ cells were pelleted, washed once in 1xPBS, incubated for 15 min at room temperature in 100μl Annexin-V-binding buffer (Annexin-V (3μl/10^6^ cells), 7AAD (eBiosciences; 4μl/10^6^ cells) in 10mM Hepes pH 7.4, 140mM NaCl, 5mM CaCl_2_). Annexin-V positive cells were quantified using a FACSCalibur flow cytometer.

### Apoptosis induction and PI3K inhibition

Apoptosis was induced in LCLs of different B-cell donors 2–3 months after infection. Cells were treated with etoposide (4μg/ml, Sigma), staurosporine (4μg/ml, Sigma) or a DMSO solvent control for 20 hrs. We also used simvastatin (2mM, Calbiochem) or an ethanol solvent control for 5 days [46]. After treatment, cells were subjected to western blotting (PARP) or Annexin-V viability staining. We used a 10nM concentration of 17β-hydroxy wortmannin (Cayman Chemical) to inhibit the PI3K in EBV wild type-infected primary B lymphocytes 14 days post-infection. Cells were treated for 17 hrs and the cell cycle profile was determined using a BrdU incorporation assay as described above.

### Western blotting

Cells were harvested, washed once in 1xPBS, lysed in RIPA buffer supplemented with a protease inhibitor cocktail (1:1000, Sigma) and sonicated. 50μg of protein were separated on a 7.5% (PARP1) or 15% (p27, BHRF1, PTEN, Actin) SDS-polyacrylamide gel and electroblotted on a protran membrane (Amersham, 0.45 NC) by wet blotting. Incubation with primary and secondary antibodies was performed as described previously [5] using primary antibodies specific for PTEN (1:8000, Abcam), BHRF1 (1:100, kindly provided by J-Y. Chen [47]), p27 (1:1000, Santa-Cruz), PARP1 (1:1000, Cell Signaling) and Actin (1:10000, Dianova).

### RNA

Total RNA was isolated using TRIzol (Life technologies, 1ml per 10^7^ B-cells) according to the manufacturers protocol. polyA+ RNA was isolated by hybridization of the total TRIzol purified RNA to oligo-dT-coupled latex beads using the nucleotrap mRNA mini isolation kit (Machery-Nagel) according to the manufacturers instructions.

Northern blots of polyA+ RNA and total RNA were performed by separating 1.5μg or 7.5μg RNA, respectively, alongside with 5μl RNA marker (0.5-10kb RNA marker, Life Technologies) on a denaturing 1% agarose/1xMOPS gel containing 2.2M formaldehyde for 5 hrs at 100V. RNA was transferred to a Hybond-XL membrane (Amersham) by capillary blotting over night in 10xSSC and cross-linked to the membrane by baking for 2 hrs at 80°C. The blot was hybridized with 50ng of a [^32^P]-α-dCTP (Perkin Elmer) radiolabeled DNA probe specific for the 3’UTR of BHRF1 (coordinates 55389:55567 in the EBV reference genome V01555.2), the BHRF1 open reading frame (coordinates 54360:54853 (V01555.2)), the BHRF1 intron (coordinates 53953:54359 (V01555.2)), or the left origin of lytic replication (coordinates 53351:53756 (V01555.2)). Labeling was obtained by using the random primed DNA labeling kit (Roche). The blot was hybridized over night at 65°C in Church buffer, washed 4 times in 0.1%SDS/1xSSC and exposed to Hyperfilm-MP (Amersham) at -80°C as indicated in the figure legends. For microRNA northern blots, 20μg of total RNA per sample was separated on 15% Mini-Protean TBE-urea acrylamide gels (BioRad) at 80V for 2 hrs in 0.5xTBE (Ambion). RNA was transferred on a Hybond N+ membrane (Amersham) by semi-dry blotting for 2,5 hrs at 250mA and UV-crosslinked to the membrane (1200μJ). The blot was hybridized with 20pmol of an [^32^P]-γ-ATP (Perkin Elmer) labeled oligonucleotide (MWG Eurofins) complementary to the seed-mutated mature miR-BHRF1-3 (TGTGCTTACACACTCAACGTTA) or the seed-mutated mature miR-BHRF1-2* of the 2/2*DSM recombinant (GCAAACGGCTGCAACAACGTTT) at 37°C for 1h in ExpressHyb solution (ClonTech). Blots were washed twice at 37°C in 2xSSC/0.05%SDS, and twice in 0.1xSSC/0.1% SDS at room temperature and exposed to Hyperfilm-MP (Amersham) at -80°C for 7 days.

### qPCR for microRNAs, BHRF1 transcripts and the Wp promoter activity

MicroRNAs were quantified using stem-loop RT qPCR [48] and miR-BHRF1 specific primers as described previously [49]. 110ng total RNA was reverse transcribed with the TaqMan miRNA reverse transcription kit (Applied Biosystems) using 12.5μM of each RT primer. Per sample, 10ng of template were mixed with 1.5μM forward primer, 0.7μM reverse primer, 0.2μM probe and TaqMan universal PCR mastermix (Applied Biosystems). The samples were incubated at 50°C for 2 min, 95°C for 10 min, followed by 40 cycles of 95°C for 15 sec and 56°C for 1 min using a StepOnePlus real-time PCR system (Applied Biosystems). All samples were measured in duplicate and the RNU48 TaqMan microRNA control assay (Applied Biosystems) was used as internal control for normalization of all samples.

To quantify different BHRF1 containing transcritps and the Wp promoter activity, 400ng total RNA was reverse transcribed using the AMV reverse transcriptase (Roche) with a mix of RT primers specific for GAPDH, combined with primers specific for the W2W1 exon junction or BHRF1 [11, 50]. qPCR was performed on 20ng of template using the TaqMan universal PCR mastermix (Applied Biosystems) and incubated at 50°C for 2 min, 95°C for 10 min, followed by 40 cycles of 95°C for 15 sec and 60°C for 1 min on a StepOnePlus real-time PCR system (Applied Biosystems). The activity of the Wp promoter and the expression of W2-BHRF1 spliced transcripts were quantified as described earlier [11, 50]. For the quantification of all BHRF1 transcripts, RNA was reverse transcribed using the universal BHRF1 RT primer [11], and amplified using 0.3μM forward primer (CCCTCTTAAT TACATTTGTG CCAGAT (54337:54362 (V01555.2)), 0.3μM reverse primer [11], and 0.2μM of the Fam-labeled probe (TAGAGCAAGA TGGCCTATTC AACAAGGGAG A (54367:54397 (V01555.2)). The VIC-labeled human GAPD (GAPDH) endogenous control (Applied Biosystems) was used as internal reference for normalization, All samples were measured in duplicate.

### Luciferase reporter assays

Oligonucleotide primers (MWG Eurofins) encoding part of the wild type 3’UTR of PTEN (coordinates 102288:102331 (NG_007466.2)) or a seed-matched mutant PTEN 3’UTR (coordinates 102288:102331 (NG_007466.2) in which the position 102311:102318 (NG_007466.2) was replaced for a stretch of eight adenines were annealed and introduced in the 3’UTR of the firefly luciferase reporter plasmid pGL4.5 (Promega), which had been modified to contain an EcoR1 and Xho1 cutting site 3’ of the luc2 open reading frame. Constructs were confirmed by sequencing.

HEK 293 cells were seeded at a density of 7*10^4^ cells per well in a 24-well cluster plate. The following day, 210ng of the wild type PTEN 3’UTR firefly luciferase fusion or of the seed-matched mutant control plasmids, and 840ng of miR-BHRF1-3 (55198:55395 (V01555.2) in pcDNA3.1(+)) or of the empty vector control pcDNA3.1(+) (Invitrogen), and 210ng of a pRL-SV40 plasmid (Promega) encoding the renilla luciferase to control for differences in cell numbers and transfection efficiency were cotransfected using 3μl metafectene per μg of plasmid DNA. The activity of the firefly and renilla luciferase were determined 2 days after transfection using the dual-luciferase reporter assay system (Promega) according to the manufactures instructions and measured using a Fluoroskan Ascent FL luminometer (Thermo Scientific) in triplicate measurements for each sample.

### Statistical analysis

GraphPad Prism 6 was used to conduct all statistical analysis. The error bars represent the standard deviation of the data sets. Statistical significance was determined using the student's t-test and all data that were derived from LCLs generated from the same blood donor were analyzed as paired samples.

## Supporting Information

S1 Fig (a) Schematic description of the recombinant EBV mutants used in this study. (b) BHRF1 protein expression in ΔBHRF1 transformed LCLs. We tested BHRF1 protein expression at day 5 in 3 pairs of LCLs transformed with the wild type virus or with ΔBHRF1 virus by western blotting using a BHRF1-specific antibody.
(DOCX)

S2 Fig miR-BHRF1 expression in cells infected with the 2/2*DSM or the 3SM recombinant virus. (a) We quantified BHRF1 microRNA expression at day 5 in cells infected with the seed-mutated recombinant viruses 2/2*DSM using RT-qPCR. Primers specific for the wild type microRNA sequences were used. (b) Processing of the seed mutated microRNA 2* was confirmed by northern blot using a radiolabeled probe complementary to the mutated miR-BHRF1-2*. (c) The expression of miR-BHRF1-1, -2, -2*, and -3 was tested at day 5 after infection with wild type virus or the miR-BHRF1-3SM recombinant virus. (d) To confirm the processing of the seed-mutated miR-BHRF1-3, northern blots were performed using a probe complementary to the mature seed-mutated miR-BHRF1-3. Arrows in (b) and (d) indicate the position of the mature miRNA, arrowheads with dotted line mark the precursor miRNA.
(DOCX)

S3 Fig PTEN expression in ΔBHRF1 recombinant infected cells. A representative western blot analysis for PTEN protein expression in wild type or ΔBHRF1 infected LCLs is shown, together with the ImageJ based quantification of 5 pairs of LCLs normalized for actin and depicted relative to the respective wt sample.
(DOCX)

S4 Fig Different BHRF1 containing transcripts were analyzed by northern blotting of total RNA and polyA+ purified RNA. (a) We performed northern blot analyses on 1.5 months old LCLs derived from the same blood sample and infected with various mutants that lack one or multiple BHRF1 miRNAs using the probes indicated in the schematic. (b and c) These figures show the results of the northern blots performed on total (b) or polyA+ (c) RNA. We used a 1 kb RNA ladder to identify the size of the different signals. We also indicate the positions of the ribosomal RNAs (.) and of the dominant RNA populations (1.3 and 2.2kb RNAs; arrow head).
(DOCX)
