# Supplementary material for: A Viral microRNA Cluster Regulates the Expression of PTEN, p27 and of a bcl-2 Homolog
Source: PLoS Pathog. 2016 Jan 22;12(1):e1005405. doi: 10.1371/journal.ppat.1005405 (PMC4723338; doi:10.1371/journal.ppat.1005405)
Supplement: S1 Fig — (b) BHRF1 protein expression in ΔBHRF1 transformed LCLs. We tested BHRF1 protein expression at day 5 in 3 pairs of LCLs transformed with the wild type virus or with ΔBHRF1 virus by western blotting using a BHRF1-specific antibody. (DOCX) [file ppat.1005405.s001.docx]

**a**

**b**
