# Supplementary material for: A Viral microRNA Cluster Regulates the Expression of PTEN, p27 and of a bcl-2 Homolog
Source: PLoS Pathog. 2016 Jan 22;12(1):e1005405. doi: 10.1371/journal.ppat.1005405 (PMC4723338; doi:10.1371/journal.ppat.1005405)
Supplement: Supplementary file 2 [file ppat.1005405.s002.docx]

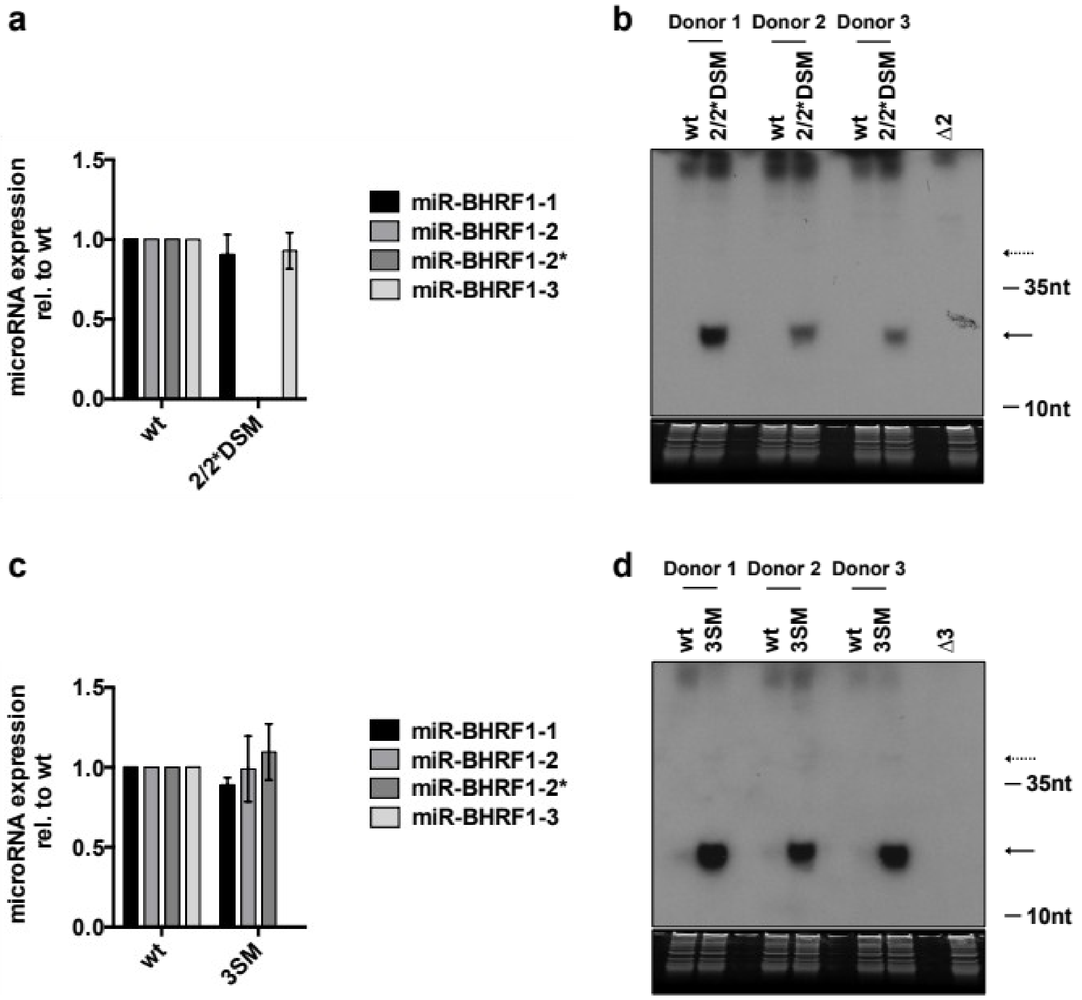
a
microRNA expression rel. to wt
1.5
1.0
0.5
0.0
wt
2/2*DSM
miR-BHRF1-1
miR-BHRF1-2
miR-BHRF1-2*
miR-BHRF1-3
b
Donor 1
Donor 2
Donor 3
wt
2/2*DSM
Δ2
35nt
10nt
c
3SM
d
Δ3
