# Supplementary material for: A Viral microRNA Cluster Regulates the Expression of PTEN, p27 and of a bcl-2 Homolog
Source: PLoS Pathog. 2016 Jan 22;12(1):e1005405. doi: 10.1371/journal.ppat.1005405 (PMC4723338; doi:10.1371/journal.ppat.1005405)
Supplement: Supplementary file 3 [file ppat.1005405.s003.docx]

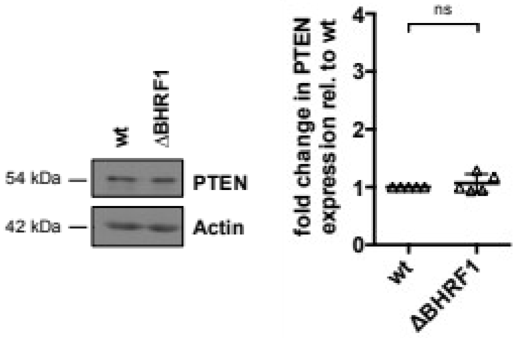

wt
ΔBHRF1
54 kDa
PTEN
42 kDa
Actin
fold change in PTEN expression rel. to wt
ns
4
3
2
1
0
wt
ΔBHRF1
