# Supplementary material for: A Viral microRNA Cluster Regulates the Expression of PTEN, p27 and of a bcl-2 Homolog
Source: PLoS Pathog. 2016 Jan 22;12(1):e1005405. doi: 10.1371/journal.ppat.1005405 (PMC4723338; doi:10.1371/journal.ppat.1005405)
Supplement: Supplementary file 4 [file ppat.1005405.s004.docx]

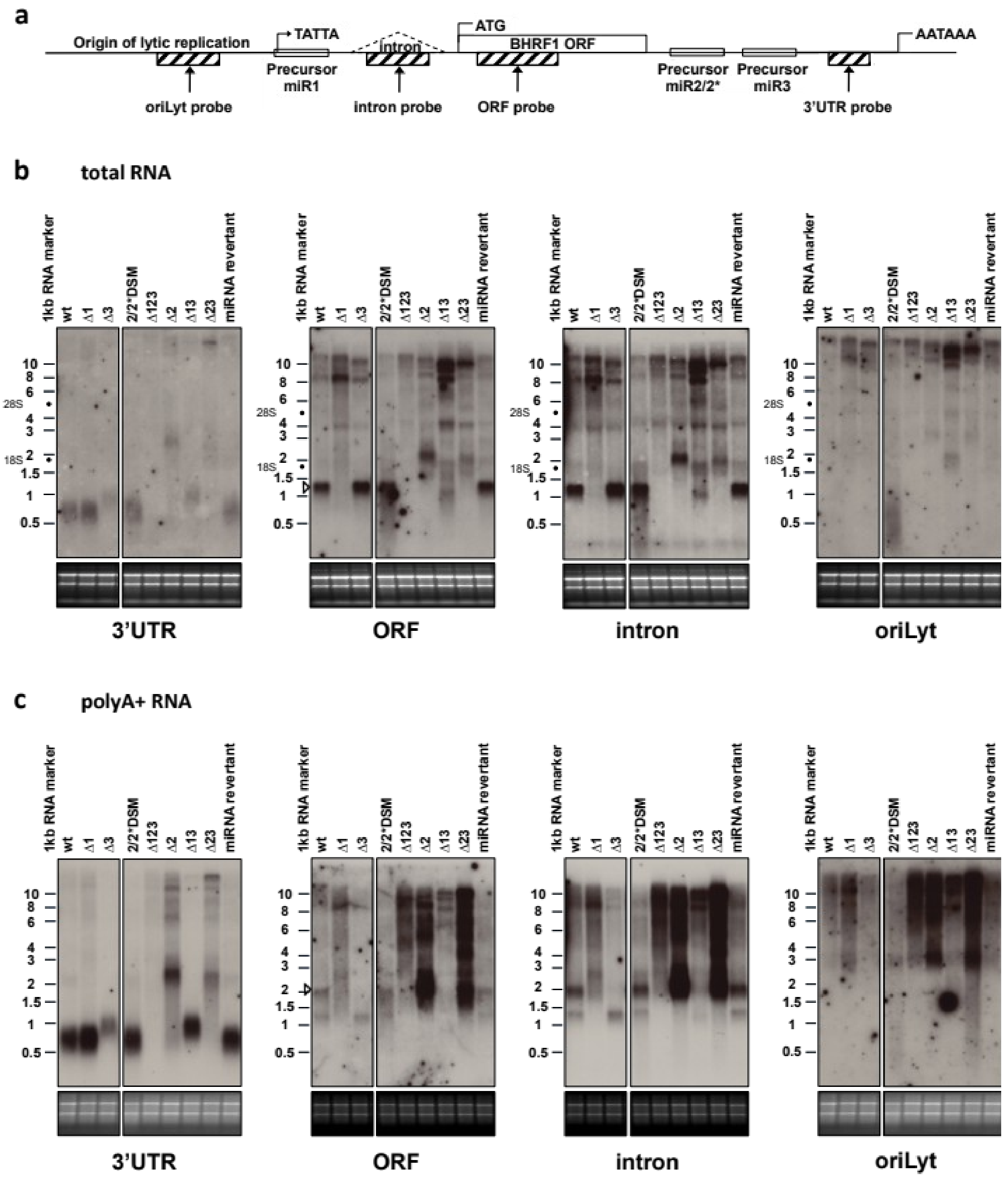
a
Origin of lytic replication
TATTA
Precursor miR1
intron
ATG
BHRF1 ORF
Precursor miR2/2*
Precursor miR3
AATAAA
oriLyt probe
intron probe
ORF probe
3'UTR probe
b
total RNA
1kb RNA marker
wt
Δ1
Δ3
2/2*DSM
Δ123
Δ2
Δ13
Δ23
miRNA revertant
10
8
6
4
3
2
1.5
1
0.5
28S
18S
3'UTR
ORF
intron
oriLyt
c
polyA+ RNA
